# Network Pharmacology and Experimental Verification Revealed the Mechanism of Yiqi Jianpi Recipe on Chronic Obstructive Pulmonary Disease

**DOI:** 10.1155/2022/8823231

**Published:** 2022-09-07

**Authors:** Ke Chen, Min Zhang, Liming Fan, Zhen Wang

**Affiliations:** ^1^The First Clinical College, Zhejiang Chinese Medical University, Hangzhou, China; ^2^Department of Pulmonary and Critical Care Medicine, The First Affiliated Hospital of Zhejiang Chinese Medical University, Hangzhou, China

## Abstract

**Objective:**

The study aimed to explore the active ingredients, targets, and mechanism of action of Yiqi Jianpi recipe (YQJPR) in the treatment of COPD based on the network pharmacology and COPD rat models.

**Methods:**

The active ingredients and targets of YQJPR were collected by TCMSP. Disease-related protein targets were obtained from GeneCards. The Venn diagram was used to show the key therapeutic targets of COPD in YQJPR. The PPI network was established by STRING, and cytoHubba plug-in was used to screen the core targets within the network. GO functional enrichment and KEGG pathway enrichment analysis were performed to describe the functions and pathways of the core targets. Cytoscape software was used to construct the ingredient-target network and the core target-enrichment pathway network. The chemical constituents of YQJPR were analyzed by HPLC-MS/MS.

**Results:**

The network pharmacology showed 152 active ingredients and 225 targets in YQJPR for the treatment of COPD. The key active ingredients were quercetin, luteolin, kaempferol, tanshinone IIA, and baicalein. The contents of quercetin and luteolin in YQJPR were quantitatively measured by HPLC-MS/MS. 22 core genes were screened, including AKT1, IL-6, JUN, VEGFA, and CASP3, which were mainly involved in BPs such as cell proliferation and differentiation, oxidative/chemical stress, and regulation of DNA-binding transcription factor activity and regulated viral infection, tumor, HIF-1, MAPK, TNF, and IL-17 pathways. Animal experiments showed that YQJPR could significantly reduce the expression of p-ERK1/2, p-Akt, c-Myc, cleaved caspase-3, and p-Stat3 in lung tissue (*p* < 0.05). HE staining showed that, compared with the model group, YQJPR significantly improved lung tissue morphology and reduced lung inflammation in rats.

**Conclusion:**

The effects of YQJPR on COPD may involve multiple components, pathways, and targets. This study provides new ideas for further and more comprehensive exploration of the therapeutic effect of YQJPR on COPD in the future.

## 1. Introduction

Chronic obstructive pulmonary disease (COPD) is currently the third leading cause of death in the world after cardiovascular disease and stroke [1], which brings a huge economic and social burden to people and seriously affects the quality of life. The World Economic Forum estimates that the global cost of COPD will reach $50 trillion per year by 2030, more than the cost of cardiovascular disease [2]. The commonly used and effective treatment methods for COPD are mainly inhaled drugs, especially bronchodilators. Nondrug treatments, such as pulmonary rehabilitation, oxygen inhalation, and other adjuvant therapy, are also effective and are current research hotspots, but their costs are high. These treatments are underutilized worldwide due to insufficient funding, resources, and patient awareness [3]. To date, no therapy has been found to slow the progression of COPD and no drug has significantly reduced its mortality or complication rates [2]. Therefore, it is necessary to explore new treatment methods to improve the quality of life of patients.

Yiqi Jianpi recipe (YQJPR) is an empirical prescription for the clinical treatment of COPD by Professor Wang, a famous Chinese medicine doctor from the Zhejiang Provincial Hospital of Traditional Chinese Medicine. It is mainly composed of Huang Qi (Radix *Astragalus*), Dang Shen (Codonopsis), Bai Zhu (Atractylodes macrocephala Koidz), Fu Lin (Poria cocos), Zhu Li (Bamboo Juice), Ban Xia (Pinelliae Rhizoma), Chen Pi (Pericarpium Citri Reticulatae), Qian Hu (Peucedani Radix), Dan Pi (Cortex Moutan), and Gan Cao (Glycyrrhizae Radix). Although YQJPR has a curative effect on COPD, its composition is complex and its pharmacodynamic mechanism is not yet clear. Therefore, there is great interest in studying active compounds and molecular targets for the treatment of COPD in YQJPR.

Network pharmacology is a recently proposed new method to study traditional Chinese medicine (TCM) by combining systematic network analysis and pharmacology to elucidate the interactions between compounds, genes, proteins and diseases [4]. Therefore, the active ingredients, targets, and mechanism of action of YQJPR in the treatment of COPD through network pharmacology, which can provide a theoretical basis for future experimental research and clinical application, were explored.

## 2. Materials and Methods

### 2.1. Screening of Active Ingredients and Targets of YQJPR

The chemical compositions of YQJPR were obtained from the TCMSP database (https://old.tcmsp-e.com/tcmsp.php). This database provides 12 pharmacokinetic properties [5], including oral bioavailability (OB), drug-likeness (DL), drug half-life (HL), and blood-brain barrier (BBB). OB is one of the most important pharmacokinetic parameters for studying oral drugs [6], and high OB is often a key indicator for determining the drug-like properties of bioactive molecules as therapeutics. It is generally considered that DL ≥ 0.18 of a compound is the selection criterion for “drug-like” compounds in traditional Chinese herbal medicine [7]. Therefore, compounds with OB ≥ 30% and DL ≥ 0.18 as active ingredients were selected for this study. Then, the targets of each herb were collected in the TCMSP database and converted into official genetic symbol through UniProt database (https://sparql.uniprot.org/).

### 2.2. Screening of Targets Related to COPD

COPD-related protein targets were provided by the GeneCard database (https://www.genecards.org/). Using “chronic obstructive pulmonary disease” as the keyword, the genes with protein-coding function and GeneCards Inferred Functionality Scores (GIFtS) ≥30 were selected.

### 2.3. Venn Diagram

The Venn diagram (made by Venny 2.1.0) was used to obtain the intersection genes of COPD and YQJPR, which were defined as “key therapeutic targets for COPD.”

### 2.4. Construction of the Ingredient-Target Network

Using the Cytoscape 3.9.1 software to link drug and disease intersection genes, the relationship between active ingredients and COPD-related targets in YQJPR was visualized.

### 2.5. Preparation of YQJPR

The preparation of YQJPR sample was performed as Dong et al. [8] and improved. Ten herbs were included in YQJPR, which were Huang Qi 30 g, Dang Shen 20 g, Bai Zhu 12 g, Fu Lin 15 g, Zhu Li Ban Xia 12 g, Chen Pi 9 g, Qian Hu 12 g, Dan Pi 12 g, and Gan Cao 3 g. All the above herbs (125 g in total) were mixed with boiling water and boiled twice (1.5 h for the first time, the amount of water was 8 times the quality of the herbs that was 1000 ml; 0.5 h for the second time, the amount of water was 6 times that was 750 ml). The two decoctions were filtered, mixed together, concentrated to 400 ml, and then frozen at −80°C overnight. After being placed in a freeze dryer for about 72 hours, the sample of YQJPR freeze-dried powder was obtained, and the powder yield was 30%. The lyophilized powder was dissolved in methanol as the test drug (namely, YQJPR) for further study. The contents of quercetin and luteolin in YQJPR were quantitatively measured by high-performance liquid chromatographic-mass spectrometry/mass spectrometry (HPLC-MS/MS). HPLC experiments were performed on an Agilent LC1290-QQQ-6470 instrument. The samples were filtered through 0.22 *μ*m membrane before injection.

#### 2.5.1. Chromatographic Conditions

HPLC separation was performed on an ACE UltraCore C18 column (2.1 × 75 mm, 2.5 *μ*m) at a column temperature of 40°C. Gradient elution was conducted with 0.1% formic acid (A) and acetonitrile (B): 0 min, 90% A; 2.5 min, 50% A; and 5 min, 10% A. The solvent flow rate was 350 *μ*L·min^−1^, and the injection volume was 3 *μ*L.

#### 2.5.2. Mass Spectrometry Conditions

The MS data were acquired in negative ionization mode and multiple reaction monitoring scan mode. Instrumental parameters were optimized as follows: capillary: 3500 V (positive) and −3000 V (negative); nozzle voltage: −1000 V (negative); source gas (nebulizer gas), 30 psi; gas temperature, 150°C; gas flow, 12 L·min^−1^; sheath gas temperature, 350°C; sheath gas flow, 12 L·min^−1^.

### 2.6. Construction of the PPI Network

Using the STRING database (https://cn.string-db.org/), the protein-protein interaction (PPI) network was established for key therapeutic targets in COPD by setting “*Homo sapiens*” in “Organism” and “moderate confidence 0.4.” The obtained PPI network data were imported into Cytoscape 3.9.1, and then, the important nodes and subnetworks in the network were screened by the cytoHubba plugin. CytoHubba predicts and explores important nodes and subnetworks in the network through several topological analysis methods [9]. In this study, two analysis methods, degree and closeness, were selected to rank nodes, select core genes, and construct subnetworks.

### 2.7. GO and KEGG Pathway Enrichment Analysis

Gene ontology (GO) analysis describes the function of genes from three aspects: biological process (BPs), cellular components (CCs), and molecular functions (MFs), and group genes with similar functions into one group [10]. Kyoto Encyclopedia of Genes and Genomes (KEGG) pathway analysis reflects the interactions of genes or gene products in pathways to predict cellular function [11].

The R packages including “colorspace,” “string,” and “ggplot2” and the Bioconductor package (https://www.bioconductor.org/) including “DOSE,” “clusterProfiler,” and “enrichplot” were used to perform GO and KEGG pathway analysis on the core genes of YQJPR and COPD [12]. According to “*p* ≤ 0.05, *q* ≤ 0.05,” the top 20 GO terms and KEGG pathways were selected. Subsequently, the data of the top 20 KEGG pathways were imported into the Cytoscape software to construct a KEGG network.

### 2.8. In Vivo Experiments in Rats

#### 2.8.1. Animals and Reagents

SD rats (male, 4–6 weeks old, 200 ± 20 g) were purchased from Zhejiang Chinese Medical University Animal Center (Grade SPF). All rats had free access to food and water and were tested in the SPF barrier laboratory of Zhejiang Chinese Medical University Animal Center. The whole protocol was approved by the Animal Ethics Committee of Zhejiang Chinese Medical University. Every effort was made to minimize the suffering of the animals during the experiment.

The main formula of YQJPR is as follows: Huang Qi, 30 g; Dang Shen, 20 g; Bai Zhu, 12 g; Fu Lin, 15 g; Zhu Li Ban Xia, 12 g; Chen Pi, 9 g; Qian Hu, 12 g; Dan Pi, 12 g; and Gan Cao 3 g. All herbs were provided by the TCM Pharmacy of the first affiliated hospital of Zhejiang Chinese Medical University (Hangzhou, China), and one dose was finally decocted to 125 ml of liquid (1 g/ml).

Hongtashan cigarettes (84-mm flue-cured cigarettes, smoke nicotine content: 0.9 mg, tar content: 10 mg, smoke carbon monoxide content: 11 mg) were provided by Hongta Tobacco Co., Ltd.

Antibody types are as follows: primary antibodies [Phospho-Akt (CST, 9271), Phospho-p44/42 MAPK (p-ERK1/2) (CST, 4370), c-Myc (CST, 5605), cleaved caspase-3 (CST, 9664), Phospho-Stat3 (CST, 9134), and GAPDH (BOSTER, BM1623)], and secondary antibodies [anti-rabbit IgG, HRP-linked antibody (CST, 7074) and anti-mouse IgG, HRP-linked Antibody (CST, 7076)].

#### 2.8.2. Grouping and Intervention

**Figure 1:**
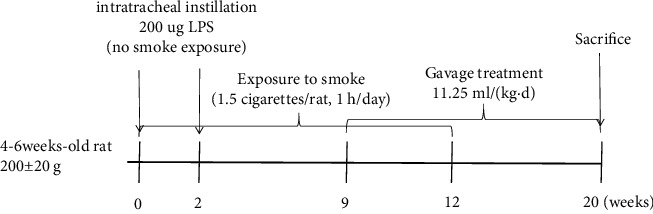
Experimental scheme diagram. In the model establishment group, 200 ug LPS was gradually instilled into the airway on days 1 and 14. These rats were not exposed to smoke on the day of instilled LPS, but were exposed to smoke for 12 weeks. From Week 9 onward, continuous drug intervention for 12 weeks was conducted and all rats (including the control group) were sacrificed and the samples were obtained at the end of week 20.

After three days of adaptive feeding, ten of the 30 SD rats were randomly selected as the control group, and the rest were selected as the model establishment group (Figure 1). Referring to previous experience [13, 14], the model establishment group (*n* = 20) used cigarette smoke exposure (CSE) combined with intratracheal instillation of LPS (Sigma) to prepare a COPD model for 12 weeks. During this period, 20 rats passively inhaled smoke (1.5 cigarettes/rat, 1 h/day) in a self-made smoking box (approximately 46 cm × 33 cm × 26 cm, made of plastic), except for the intratracheal instillation of 0.2 ml (200 ug) LPS (no smoke exposure) on days 1 and 14. From Week 9 onward, the model establishment group was randomly divided into the model group (*n* = 10) and the YQJPR group (*n* = 10). The model group continued to passively smoke until the end of 12 weeks, and the YQJPR group started TCM intervention and continued to passively smoke 1 hour after gavage. The YQJPR group continued the TCM intervention after the 12th week of modelling, and the whole TCM intervention lasted for 12 weeks. The dose of YQJPR to rats was calculated from the dose conversion method between humans and rats [15]. The adult clinical dosage of YQJPR was 1.786 g/(kg·d), and then, the YQJPR group was given 11.25 g/(kg·d) by gavage, while the control and model groups were given the same amount of normal saline by gavage. After treatment, all rats were fasted overnight and anesthetized by intraperitoneal injection of 2.5% sodium pentobarbital (0.3 ml/100 g) the next day. The right part of the lung tissue was stored at −80°C for western blot, and the left part was fixed with 4% paraformaldehyde for HE staining to observe the morphological structure of the lung tissue.

#### 2.8.3. Western Blot

The lung tissue was homogenized with RIPA lysis buffer (Boster, Wuhan, China) and centrifuged (10,000 × *g*, 10 min, 4°C) to collect total protein. After denaturation with SDS-PAGE Sample Loading Buffer (Beyotime, Shanghai, China), proteins were separated by 10% or 12% SDS-PAGE and transferred to PVDF membranes (Boster, Wuhan, China). The membranes were blocked with 5% nonfat dry milk (Dawen Biotec, Hangzhou, China) at room temperature. After 1.5 h, they were rinsed with TBST and incubated overnight at 4°C with primary antibodies against p-ERK1/2 (1 : 1000, anti-rabbit), p-Akt (1 : 1000, anti-rabbit), c-Myc (1 : 500, anti-rabbit), cleaved caspase-3 (1 : 1000, anti-rabbit), p-Stat3 (1 : 500, anti-rabbit), and GAPDH (1 : 5000, anti-mouse). Then, the blots were washed with TBST (10 min*∗*3). The blots were subsequently incubated with a secondary antibody (1 : 1000, goat anti-rabbit or goat anti-mouse antibody) at room temperature for 1.5 h. Finally, the blots were washed with TBST (10 min*∗*3) and visualized with enhanced chemiluminescence reagent (Fdbio science, Hangzhou, China) and analyzed with Bio-Rad Machine (USA).

ImageJ software (version 1.8.0) analyzed the gray value of the blots and took the ratio with the gray value of GAPDH as the relative expression level, and then, the ratio was normalized.

#### 2.8.4. Statistical Analysis

GraphPad Prism 9 was used for statistics. All experimental data were obtained from at least three repeated independent trials and expressed as mean ± SD. Statistical differences between the groups were identified by one-way ANOVA. *p* < 0.05 indicates a statistical difference.

## 3. Results

### 3.1. Active Ingredient-Target Analysis

Searched in the TCMSP database and screened with OB ≥ 30% and DL ≥ 0.18 as the condition, a total of 208 active ingredients of YQJPR were obtained, including 20 in Radix *Astragalus*, 21 in Codonopsis, 7 in Atractylodes macrocephala Koidz, 15 in Poria cocos, 13 in Pinelliae Rhizoma, 5 in Pericarpium Citri Reticulatae, 24 in Peucedani Radix, 11 in Cortex Moutan, and 92 in Glycyrrhizae Radix. Bamboo juice was filtered out during this screening process (Supplementary Table 1). The targets of each herb were collected, screened, and normalized by the TCMSP and UniProt databases. A total of 2926 drug targets were obtained after removing duplicate targets, which were summarized by drug-ingredient-target correspondence.

### 3.2. Acquisition and Selection of COPD-Related Targets

With “chronic obstructive pulmonary disease” as the keyword, the genes with protein-coding function and GIFtS ≥30 were selected as the targets in the GeneCards database, and 5057 targets were finally collected.

### 3.3. Venn Diagram

**Figure 2:**
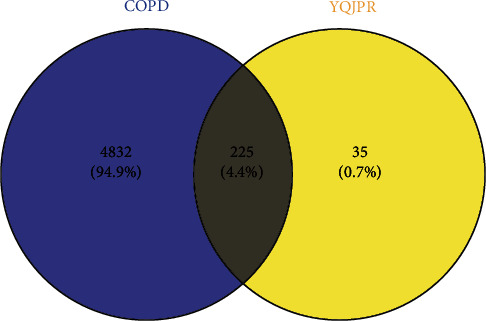
Venn diagram of intersection genes related to COPD and YQJPR. Blue indicates targets associated with COPD, and yellow indicates targets associated with YQJPR.

We constructed Venn diagrams (Figure 2) by Venny 2.1.0 to depict the intersection targets of COPD and YQJPR (the key therapeutic targets for COPD). The analysis revealed that there were 225 targets related to both COPD and YQJPR (Supplementary Table 2).

### 3.4. Ingredient-Target Network Analysis

**Figure 3:**
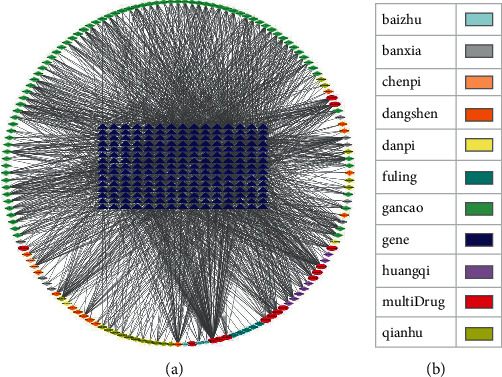
Ingredient-target network and annotation. (a) Ingredient-target network. Target genes are in the middle, and active ingredients are in the periphery. (b) Network notation. Different colors represent different drugs except blue, which represents genes.

The ingredient-target network (Figure 3) was constructed by the Cytoscape 3.9.1 software to visualize the relationship between 152 active ingredients and 225 targets. The network included 377 nodes and 1931 edges. These edges indicated interactions between ingredients and targets. The active ingredients and the targets with greater correlation were screened by the cytoHubba plug-in. The degree value represented the number of edges associated with the node. The greater the degree value, the greater the correlation between the ingredient and the target.

The results showed that in this network (Table 1), MOL000098 (quercetin), MOL000006 (luteolin), MOL000422 (kaempferol), MOL007154 (tanshinone IIA), MOL000378 (7-O-methylisomucronulatol), MOL003896 (7-methoxy-2-methyl isoflavone), and MOL002714 (baicalein) were associated with at least 30 targets, and PTGS2, ESR1, HSP90AA1, AR, NOS2, PPARG, NCOA2, PRSS1, SCN5A, CDK2, PTGS1, and GSK3B were regulated by at least 60 compounds.

### 3.5. Quality Evaluation of YQJPR

**Figure 4:**
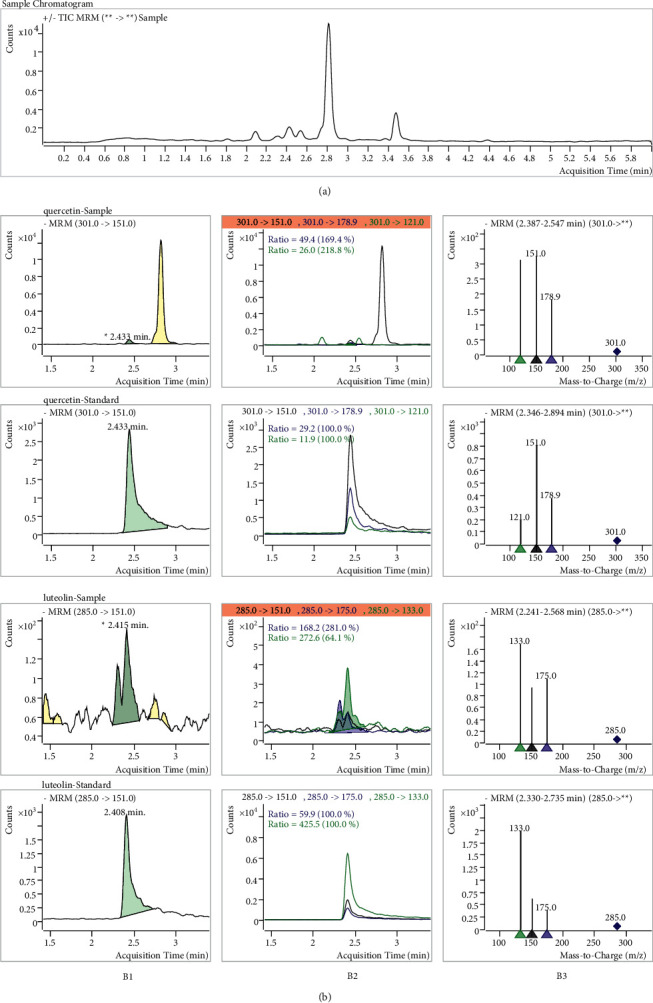
HPLC-MS/MS results of the sample and reference substances. (a): sample chromatogram. B1: MRM chromatograms for quantitative detection conditions. B2: MRM chromatograms. B3: secondary mass spectrum.

The HPLC-MS/MS parameters of the standards after optimization are shown in Table 2. Two key compounds (quercetin and luteolin) in YQJPR were well identified by comparing their retention times and mass spectra with the standards by using HPLC-MS/MS analysis (Figure 4), whose contents in YQJPR freeze-dried powder were 70.2 and 60.9 ng/g. This result was calculated by quantitative analysis.

### 3.6. PPI Network Construction

**Figure 5:**
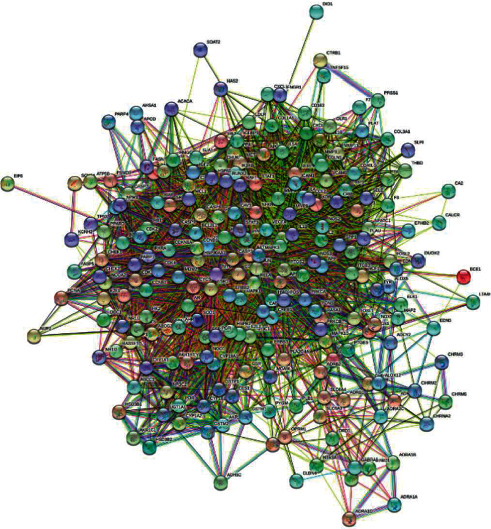
PPI network for COPD and YQJPR. Line color indicates the type of interaction evidence.

The PPI network (Figure 5) was constructed from the same 225 key therapeutic targets for COPD, including 225 nodes and 4224 edges, with the average node degree of 37.5 and the average local clustering coefficient of 0.594.

**Figure 6:**
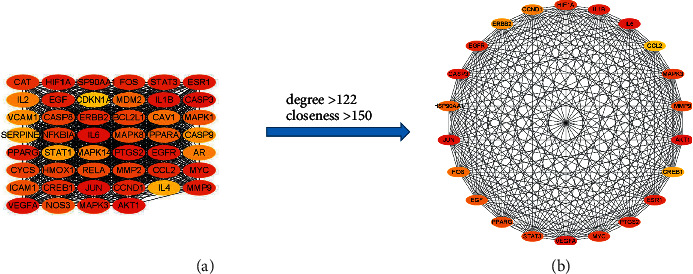
Construction of a subnetwork of 22 core genes in the PPI network. (a) Top 46 targets of degree in the PPI network (degree > 122). (b) A subnetwork of 22 core genes (degree > 122 and closeness > 150). From red to yellow, the degree value and the closeness value decrease.

The nodes of the PPI network were calculated by the cytoHubba plug-in and were ranked according to degree and closeness. The top 22 targets with “degree > 122, closeness > 150” in the PPI network were selected (Figure 6(a)), which were AKT1, IL-6, JUN, VEGFA, CASP3, IL-1B, ESR1, PTGS2, EGFR, MYC, HIF1A, MAPK3, STAT3, etc. These targets were suggested to be the core genes of YQJPR in treating COPD, and then, a subnetwork (Figure 6(b)) was constructed for the core genes.

### 3.7. GO and KEGG Analysis

GO and KEGG enrichment analysis of 22 core genes was performed to determine the underlying molecular mechanism of YQJPR in the treatment of COPD. Results for “*p* ≤ 0.05, *q* ≤ 0.05” were retained, and bubble charts were drawn for the top 20 clearly enriched entries.

#### 3.7.1. GO Analysis

**Figure 7:**
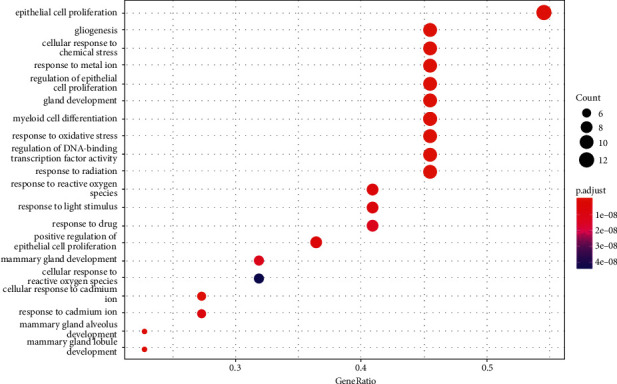
GO analysis of the core genes of YQJPR and COPD. Top 20 BPs with the most enriched core genes (*p* < 0.05). The size of the spot represents the number of genes enriched, and the shade of color represents the adjusted *p* value.

The top 20 BPs with the most enriched core genes were analyzed (Table 3 and Figure 7).

#### 3.7.2. KEGG Pathway Analysis

**Figure 8:**
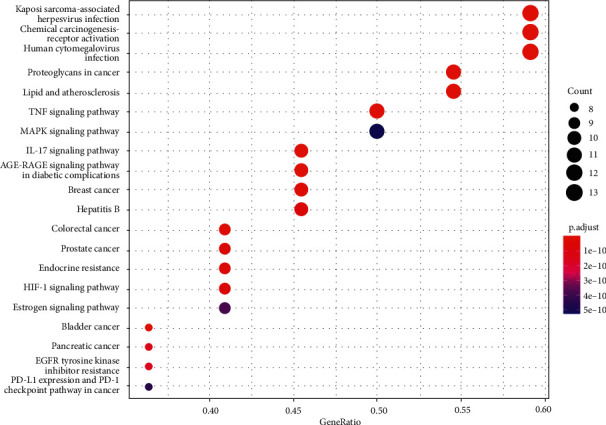
KEGG pathway analysis of the core genes of YQJPR and COPD. Top 20 pathways with the most enriched core genes (*p* < 0.05). The size of the spot represents the number of genes enriched, and the shade of color represents the adjusted *p* value.

KEGG analysis showed that the relevant YQJPR signaling pathways in the treatment of COPD included virus infection, TNF, IL-17, HIF-1, MAPK, AGE-RAGE, and cancer pathways (Supplementary Table 3). The top 20 pathways with the most enriched core genes for the treatment of COPD are selected in Figure 8.

#### 3.7.3. KEGG Network Analysis

**Figure 9:**
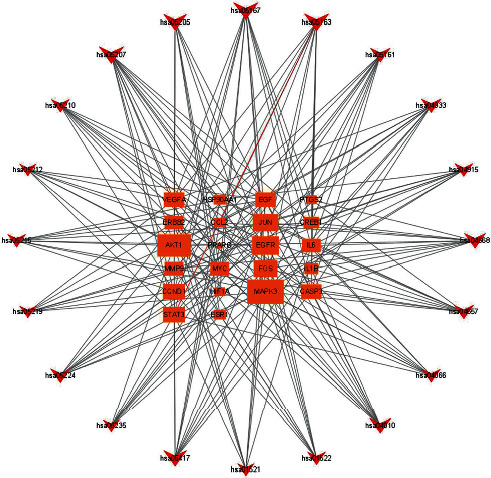
KEGG network (top-20 KEGG pathways and enriched genes). Peripheral red represents the KEGG pathway, and the middle orange represents core genes. The more significant the enrichment, the larger the shape.

The KEGG network was constructed to visualize the relationship between the top 20 pathways and enriched genes through the Cytoscape software (Figure 9). The results showed that MAPK3, AKT1, EGFR, JUN, FOS, STAT3, CCND1, VEGF, CASP3, and EGF were associated with at least 10 different pathways.

### 3.8. Experimental Verification

#### 3.8.1. Histopathological Examination

**Figure 10:**
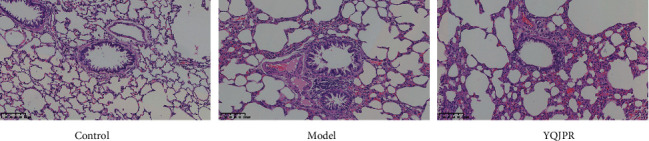
HE staining of rat lung tissue to confirm the successful establishment of the COPD rat model (×20).

To determine whether the COPD model was established successfully, HE staining of rat lung tissue was performed, mainly to observe the structure of the trachea and alveoli.  Figure 10 shows that the COPD rat model was successfully established. In the control group, the lung tissue structure was intact, and there was no obvious inflammatory cell infiltration in the alveolar cavity. Compared with the control group, the model group showed alveolar expansion and fusion and obvious inflammatory cell infiltration around the trachea. Compared with the model group, the lung tissue morphology of the YQJPR group was improved, and the peritracheal inflammatory infiltration was significantly reduced.

#### 3.8.2. Western Blot of Lung Tissue

**Figure 11:**
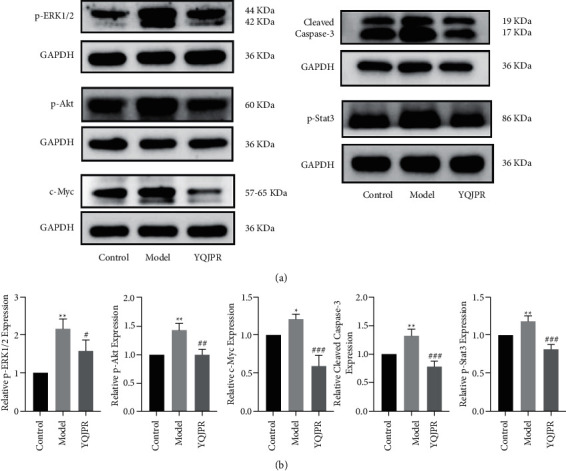
Effects of YQJPR on the levels of p-ERK1/2, p-Akt, c-Myc, cleaved caspase-3, and p-Stat3 in lung tissue of COPD rats. (a) Representative protein bands of western blot. (b) Quantitative result. The data show mean ± SD, with at least three independent replicates. ^*∗*^*p* < 0.05 and ^*∗∗*^*p* < 0.01 compared with the control group; ^#^*p* < 0.05, ^##^*p* < 0.01, and ^###^*p* < 0.001 compared with the model group.

In order to identify the results of the network pharmacology analysis, five targets were selected: MAPK3, AKT1, MYC, STAT3, and CASP3 for protein validation (Figure 11).

The results showed that compared with the control group, the expressions of p-ERK1/2, p-Akt, c-Myc, cleaved caspase-3, and p-Stat3 in the lung tissue of the model group were significantly increased. Compared with the model group, YQJPR significantly inhibited the expressions of p-ERK1/2, p-Akt, c-Myc, cleaved caspase-3, and p-Stat3.

## 4. Discussion

COPD is a preventable and treatable progressive inflammatory disease of the airway, alveoli, and microvascular system characterized by respiratory symptoms with incomplete and persistent airflow restriction [16]. The pathogenesis of COPD is complex, and the existing treatments are difficult to meet expectations, so it is urgent to explore new treatment methods. YQJPR is an empirical prescription with decades of clinical experience by Professor Wang, a famous Chinese medicine doctor from Zhejiang. YQJPR has unique advantages in the treatment of COPD, but it is difficult to popularize and use due to the complex ingredients of the TCM compound. Network pharmacology provides a new approach for the research of the pharmacological mechanism of TCM, and we try to use it to predict the components, targets, and mechanisms of the TCM compounds.

In this study, 152 ingredients and 225 targets of YQJPR related to COPD were identified, indicating that YQJPR is a treatment of COPD through combined regulation of multiple ingredients and multiple targets. Among them, quercetin, luteolin, kaempferol, tanshinone IIA, and baicalein regulated at least 30 targets and were considered as key active ingredients. And it was verified that quercetin and luteolin did exist in YQJPR by HPLC-MS/MS. The above five compounds are all flavonoids with various pharmacological effects, which include antibacterial, anti-inflammatory, antioxidant, and anticancer. Studies have shown that a diet rich in quercetin can reduce the risk of asthma, bronchial hyperresponsiveness, and COPD [17, 18]. *In vitro* and *in vivo* experiments have shown that quercetin improves lung function in COPD mice and significantly reduces oxidative stress, lung inflammation, and goblet cell metaplasia induced by cigarette smoke exposure [19, 20]. Luteolin can modulate multiple signaling pathways (such as NF-*κ*B, JAK-STAT, and TLR) and inhibit inflammatory factors (such as IL-1*β*, IL-6, IL-8, IL-17, IL-22, TNF-*α*, and COX-2) to exert anti-inflammatory effects [21]. Luteolin can prevent NF-*κ*B pathway activation and inhibit apoptosis and inflammation *in vitro* and *in vivo* by downregulating miR-132 [22]. Kaempferol can induce apoptosis and downregulate the expression of AKT/PI3K phosphorylation, ERK pathway, and MAPK, and inhibit the growth of A549 cells in a concentration-dependent manner [23]. Tanshinone IIA inhibits inflammation and oxidative stress induced by cigarette smoke by blocking MAPK/HIF-1*α* pathway, effectively alleviating the progression of COPD [24, 25]. Baicalein mainly has anti-inflammatory and anticancer effects and can treat pneumonia caused by the streptococcus *pneumoniae* infection by reducing the permeability of the alveolar capillary membrane, inhibiting pulmonary inflammatory response and apoptosis [26]. Baicalein can also significantly reduce hemodynamic parameters, inhibit pulmonary remodeling, reduce apoptosis and inflammation, and effectively improve lung injury in pulmonary hypertension model [27].

Through constructing PPI network of intersection targets between YQJPR and COPD, 22 core genes, including AKT1, IL-6, JUN, VEGFA, CASP3, IL-1B, PTGS2, MYC, MAPK3, and STAT3, were identified. IL-1 and IL-6 are both secreted proteins associated with inflammation and immune responses, with IL-1 mainly being produced by pathogens that stimulate cells of the innate immune system, such as monocytes and macrophages. IL-1 is highly expressed in small airway epithelial cells, which inhibits CSE-induced cell proliferation and promotes apoptosis, but can also promote inflammation and aggravate cell damage [28, 29]. IL-6 is a multifunctional cytokine with various roles in immunity, development, metabolism, aging, and cancer and can stimulate B cells to produce antibodies [30]. Jun and Fos are the main components of transcriptional activator protein 1 (AP-1), which are closely related to cell proliferation, apoptosis, and inflammatory response [31–33]. Caspase-3 is a key executor of apoptosis [34], and STAT3 is a signaling protein closely related to cell growth, differentiation, and survival [35]. MAPKs are a family of serine/threonine protein kinases that can regulate various extracellular stimulus signals through four linear cascades composed of MAPKs to regulate corresponding cellular processes, in which ERK1/2 (MAPK1/3) cascade preferentially regulates proliferation, differentiation, and migration [36]. MYC is a multifunctional transcription factor that can regulate the expression of many genes involved in biological processes such as cell growth, proliferation, apoptosis, and metabolism [37]. COX2 (encoded by PTGS2) is a product of multiple stimuli, linked to inflammation and tumor cell growth [38]. It is hardly expressed in normal cells but is expressed in highly proliferating cells. COX2 can activate macrophages or other inflammatory cells to transform the inflammatory site into a precancerous microenvironment, which has carcinogenic effects [39].

To understand the molecular mechanism of YQJPR in the treatment of COPD, GO analysis and KEGG enrichment analysis of 22 core genes were performed. GO analysis revealed that core target genes were strongly associated with the following BPs: cell proliferation and differentiation, oxidative/chemical stress, glandular development, regulation of DNA-binding transcription factor activity, and response to metal ions. The analysis of the KEGG pathway was primarily related to TNF, IL-17, HIF-1, MAPK, AGE-RAGE, virus infection, and cancer signaling pathways. MAPK is mainly involved in cell proliferation, differentiation, and immune regulation. It has been shown that the p38 MAPK pathway can be activated by CSE to stimulate pro-inflammatory cytokine expression [40]. Studies have found that the TNF and IL-17 signaling pathways play important roles in the inflammatory response. TNF-*α*, the most important cytokine in the TNF pathway, is elevated in COPD and can induce the release of other pro-inflammatory factors [29]. IL-17A exacerbates inflammation and mucus production in the pathogenesis of COPD in an autocrine way [41]. IL-17, a signature cytokine secreted by Th17 cells, plays a major role in the IL-17 pathway in a pro-inflammatory manner. It controls inflammation by regulating the expression of inflammatory genes in cells in most nonhematopoietic organs, such as IL-1, IL-6, IL-8, TNF, CCL2, and MMP1/3/9/13 [42]. The pulmonary circulation is chronically hypoxic in COPD [43], leading to HIF-1 signaling pathway activation and upregulated HIF-1*α* expression. The HIF-1 pathway is a major regulator of angiogenesis, an adaptive response to tissue hypoxia [44, 45]. HIF-1*α* accumulation directly upregulates VEGF levels, which contribute to airway and vascular remodeling, to improve the oxygen supply to the pulmonary circulation [46]. The results suggest that YQJPR may act on the target genes to treat COPD through the above-mentioned pathways.

Western blotting was used to verify five key targets (MAPK3, AKT1, MYC, STAT3, and CASP3) that were predicted in the PPI network. According to the results of the BPs and the KEGG analysis, these five genes were mainly enriched in the virus infection, cancer, MAPK, HIF-1, TNF, and IL-17 signaling pathways. It is closely related to biological processes such as cell proliferation and differentiation, oxidative/chemical stress, response to metal ion, and regulation of DNA-binding transcription factor activity. The results showed that compared with the model group, YQJPR significantly downregulated the expression of p-ERK1/2, p-Akt, c-Myc, cleaved caspase-3, and p-Stat3 in lung tissue. Therefore, the molecular mechanism of YQJPR treatment of COPD has been more clearly verified.

## 5. Conclusions

In this study, the key active ingredients, key targets, and pathways of YQJPR in the treatment of COPD were selected by network pharmacology. The results show that YQJPR can interfere with the development of COPD through multiple ingredients, targets, and pathways. In conclusion, YQJPR effectively improved lung inflammation in COPD rats. Especially, quercetin, luteolin, kaempferol, tanshinone IIA, and baicalein were the main effectors in YQJPR, which primarily downregulated the expressions of five key targets (MAPK3, AKT1, MYC, STAT3, and CASP3), and subsequently inhibited the activation of virus infection, cancer, MAPK, HIF-1, TNF, and IL-17 pathways, thereby alleviating CS/LPS-induced lung inflammation.

The disadvantage of this study is that only one detection method was used in the COPD rat model, and the pathways identified above were not verified, so further in-depth and comprehensive studies are needed. This study provides ideas and a basis for exploring the therapeutic effect of YQJPR on COPD in the future.

## Figures and Tables

**Table 1:** The degree value of the main nodes in the ingredient-target network.

Node name	Compound name	Degree
*(a) Main active ingredients (Degree > 30)*		
MOL000098	Quercetin	126
MOL000006	Luteolin	51
MOL000422	Kaempferol	51
MOL007154	Tanshinone iia	38
MOL000378	7-O-Methylisomucronulatol	35
MOL003896	7-Methoxy-2-methyl isoflavone	34
MOL002714	Baicalein	32

*(b) Main targets (Degree > 60)*		
PTGS2		123
ESR1		90
HSP90AA1		86
AR		77
NOS2		70
PPARG		68
NCOA2		66
PRSS1		64
SCN5A		63
CDK2		63
PTGS1		61
GSK3B		61

**Table 2:** Retention time and HPLC-MS/MS parameters of the standards.

Compound name	Precursor ion (−)	Product ion (−)	Collision energy	tR (min)
Quercetin	301	178.9/151/121	23	2.433
Luteolin	285	175/151/133	30	2.408

**Table 3:** Top 20 biological processes of core genes' enrichment based on GO analysis.

Id	Description	*p*. adjust	geneID
GO: 0050673	Epithelial cell proliferation	1.81E-11	AKT1/IL-6/VEGFA/ESR1/EGFR/MYC/HIF1A/PPARG/EGF/CCND1/ERBB2/CCL2
GO: 0042063	Gliogenesis	2.93E-10	AKT1/IL-6/IL-1B/EGFR/MAPK3/STAT3/PPARG/ERBB2/CREB1/CCL2
GO: 0062197	Cellular response to chemical stress	1.03E-09	AKT1/IL-6/JUN/CASP3/EGFR/HIF1A/MAPK3/MMP9/PPARG/FOS
GO: 0010038	Response to metal ion	1.03E-09	AKT1/JUN/CASP3/EGFR/HIF1A/MAPK3/MMP9/FOS/CCND1/CREB1
GO: 0050678	Regulation of epithelial cell proliferation	1.34E-09	AKT1/VEGFA/EGFR/MYC/HIF1A/PPARG/EGF/CCND1/ERBB2/CCL2
GO: 0048732	Gland development	2.50E-09	AKT1/IL-6/VEGFA/ESR1/EGFR/HIF1A/MAPK3/EGF/CCND1/CREB1
GO: 0030099	Myeloid cell differentiation	2.62E-09	JUN/VEGFA/CASP3/MYC/HIF1A/STAT3/MMP9/PPARG/FOS/CREB1
GO: 0006979	Response to oxidative stress	3.90E-09	AKT1/IL-6/JUN/CASP3/PTGS2/EGFR/HIF1A/MAPK3/MMP9/FOS
GO: 0051090	Regulation of DNA-binding transcription factor activity	3.90E-09	AKT1/IL-6/JUN/VEGFA/IL-1B/ESR1/MAPK3/STAT3/PPARG/FOS
GO: 0009314	Response to radiation	3.90E-09	AKT1/JUN/CASP3/EGFR/MYC/HIF1A/MMP9/FOS/CCND1/CREB1
GO: 0000302	Response to reactive oxygen species	1.03E-09	AKT1/IL-6/JUN/CASP3/EGFR/HIF1A/MAPK3/MMP9/FOS
GO: 0009416	Response to light stimulus	5.93E-09	AKT1/CASP3/EGFR/MYC/HIF1A/MMP9/FOS/CCND1/CREB1
GO: 0042493	Response to drug	1.34E-08	JUN/CASP3/IL-1B/EGFR/MYC/PPARG/FOS/CCND1/CREB1
GO: 0050679	Positive regulation of epithelial cell proliferation	5.93E-09	AKT1/VEGFA/EGFR/MYC/HIF1A/EGF/CCND1/ERBB2
GO: 0030879	Mammary gland development	1.31E-08	AKT1/VEGFA/ESR1/HIF1A/EGF/CCND1/CREB1
GO: 0034614	Cellular response to reactive oxygen species	4.37E-08	AKT1/IL-6/JUN/EGFR/MAPK3/MMP9/FOS
GO: 0071276	Cellular response to cadmium ion	1.16E-09	AKT1/JUN/EGFR/MAPK3/MMP9/FOS
GO: 0046686	Response to cadmium ion	8.07E-09	AKT1/JUN/EGFR/MAPK3/MMP9/FOS
GO: 0060749	Mammary gland alveolus development	1.94E-09	VEGFA/ESR1/HIF1A/EGF/CCND1
GO: 0061377	Mammary gland lobule development	1.94E-09	VEGFA/ESR1/HIF1A/EGF/CCND1

## Data Availability

The data used to support the findings of this study are available from the corresponding author upon request.

## Supplementary Materials

Supplementary Materials Supplementary Table 1: information of collected YQJPR active ingredients. Supplementary Table 2: results of Venn diagram. Supplementary Table 3: data of KEGG enrichment analysis.
