# Supplementary material for: Network Pharmacology and Experimental Verification Revealed the Mechanism of Yiqi Jianpi Recipe on Chronic Obstructive Pulmonary Disease
Source: Evid Based Complement Alternat Med. 2022 Sep 7;2022:8823231. doi: 10.1155/2022/8823231 (PMC9473879; doi:10.1155/2022/8823231)
Supplement: Supplementary Materials — Supplementary Table 1: information of collected YQJPR active ingredients. Supplementary Table 2: results of Venn diagram. Supplementary Table 3: data of KEGG enrichment analysis. [file 8823231.f1.zip › Supplementary Table 1.docx]

**Supplementary Table 1: information of collected YQJPR active ingredients.**

| **Ingredients of Huangqi.** | |  |  |
| --- | --- | --- | --- |
| **Mol ID** | **Molecule name** | **OB(%)** | **DL** |
| MOL000211 | Mairin | 55.38 | 0.78 |
| MOL000239 | Jaranol | 50.83 | 0.29 |
| MOL000296 | hederagenin | 36.91 | 0.75 |
| MOL000033 | (3S,8S,9S,10R,13R,14S,17R)-10,13-dimethyl-17-[(2R,5S)-5-propan-2-yloctan-2-yl]-2,3,4,7,8,9,11,12,14,15,16,17-dodecahydro-1H-cyclopenta[a]phenanthren-3-ol | 36.23 | 0.78 |
| MOL000354 | isorhamnetin | 49.6 | 0.31 |
| MOL000371 | 3,9-di-O-methylnissolin | 53.74 | 0.48 |
| MOL000374 | 5'-hydroxyiso-muronulatol-2',5'-di-O-glucoside | 41.72 | 0.69 |
| MOL000378 | 7-O-methylisomucronulatol | 74.69 | 0.3 |
| MOL000379 | 9,10-dimethoxypterocarpan-3-O-尾-D-glucoside | 36.74 | 0.92 |
| MOL000380 | (6aR,11aR)-9,10-dimethoxy-6a,11a-dihydro-6H-benzofurano[3,2-c]chromen-3-ol | 64.26 | 0.42 |
| MOL000387 | Bifendate | 31.1 | 0.67 |
| MOL000392 | formononetin | 69.67 | 0.21 |
| MOL000398 | isoflavanone | 109.99 | 0.3 |
| MOL000417 | Calycosin | 47.75 | 0.24 |
| MOL000422 | kaempferol | 41.88 | 0.24 |
| MOL000433 | FA | 68.96 | 0.71 |
| MOL000438 | (3R)-3-(2-hydroxy-3,4-dimethoxyphenyl)chroman-7-ol | 67.67 | 0.26 |
| MOL000439 | isomucronulatol-7,2'-di-O-glucosiole | 49.28 | 0.62 |
| MOL000442 | 1,7-Dihydroxy-3,9-dimethoxy pterocarpene | 39.05 | 0.48 |
| MOL000098 | quercetin | 46.43 | 0.28 |

| **Ingredients of Dangshen.** | |  |  |
| --- | --- | --- | --- |
| **Mol ID** | **Molecule name** | **OB(%)** | **DL** |
| MOL001006 | poriferasta-7,22E-dien-3beta-ol | 42.98 | 0.76 |
| MOL002140 | Perlolyrine | 65.95 | 0.27 |
| MOL002879 | Diop | 43.59 | 0.39 |
| MOL003036 | ZINC03978781 | 43.83 | 0.76 |
| MOL000449 | Stigmasterol | 43.83 | 0.76 |
| MOL003896 | 7-Methoxy-2-methyl isoflavone | 42.56 | 0.2 |
| MOL004355 | Spinasterol | 42.98 | 0.76 |
| MOL004492 | Chrysanthemaxanthin | 38.72 | 0.58 |
| MOL005321 | Frutinone A | 65.9 | 0.34 |
| MOL000006 | luteolin | 36.16 | 0.25 |
| MOL006554 | Taraxerol | 38.4 | 0.77 |
| MOL006774 | stigmast-7-enol | 37.42 | 0.75 |
| MOL007059 | 3-beta-Hydroxymethyllenetanshiquinone | 32.16 | 0.41 |
| MOL007514 | methyl icosa-11,14-dienoate | 39.67 | 0.23 |
| MOL008391 | 5alpha-Stigmastan-3,6-dione | 33.12 | 0.79 |
| MOL008393 | 7-(beta-Xylosyl)cephalomannine_qt | 38.33 | 0.29 |
| MOL008397 | Daturilin | 50.37 | 0.77 |
| MOL008400 | glycitein | 50.48 | 0.24 |
| MOL008406 | Spinoside A | 39.97 | 0.4 |
| MOL008407 | (8S,9S,10R,13R,14S,17R)-17-[(E,2R,5S)-5-ethyl-6-methylhept-3-en-2-yl]-10,13-dimethyl-1,2,4,7,8,9,11,12,14,15,16,17-dodecahydrocyclopenta[a]phenanthren-3-one | 45.4 | 0.76 |
| MOL008411 | 11-Hydroxyrankinidine | 40 | 0.66 |

| **Ingredients of Baizhu.** | |  |  |
| --- | --- | --- | --- |
| **Mol ID** | **Molecule name** | **OB(%)** | **DL** |
| MOL000020 | 12-senecioyl-2E,8E,10E-atractylentriol | 62.4 | 0.22 |
| MOL000021 | 14-acetyl-12-senecioyl-2E,8E,10E-atractylentriol | 60.31 | 0.31 |
| MOL000022 | 14-acetyl-12-senecioyl-2E,8Z,10E-atractylentriol | 63.37 | 0.3 |
| MOL000028 | α-Amyrin | 39.51 | 0.76 |
| MOL000033 | (3S,8S,9S,10R,13R,14S,17R)-10,13-dimethyl-17-[(2R,5S)-5-propan-2-yloctan-2-yl]-2,3,4,7,8,9,11,12,14,15,16,17-dodecahydro-1H-cyclopenta[a]phenanthren-3-ol | 36.23 | 0.78 |
| MOL000049 | 3β-acetoxyatractylone | 54.07 | 0.22 |
| MOL000072 | 8β-ethoxy atractylenolide Ⅲ | 35.95 | 0.21 |

| **Ingredients of Fulin.** | |  |  |
| --- | --- | --- | --- |
| **Mol ID** | **Molecule name** | **OB(%)** | **DL** |
| MOL000273 | (2R)-2-[(3S,5R,10S,13R,14R,16R,17R)-3,16-dihydroxy-4,4,10,13,14-pentamethyl-2,3,5,6,12,15,16,17-octahydro-1H-cyclopenta[a]phenanthren-17-yl]-6-methylhept-5-enoic acid | 30.93 | 0.81 |
| MOL000275 | trametenolic acid | 38.71 | 0.8 |
| MOL000276 | 7,9(11)-dehydropachymic acid | 35.11 | 0.81 |
| MOL000279 | Cerevisterol | 37.96 | 0.77 |
| MOL000280 | (2R)-2-[(3S,5R,10S,13R,14R,16R,17R)-3,16-dihydroxy-4,4,10,13,14-pentamethyl-2,3,5,6,12,15,16,17-octahydro-1H-cyclopenta[a]phenanthren-17-yl]-5-isopropyl-hex-5-enoic acid | 31.07 | 0.82 |
| MOL000282 | ergosta-7,22E-dien-3beta-ol | 43.51 | 0.72 |
| MOL000283 | Ergosterol peroxide | 40.36 | 0.81 |
| MOL000285 | (2R)-2-[(5R,10S,13R,14R,16R,17R)-16-hydroxy-3-keto-4,4,10,13,14-pentamethyl-1,2,5,6,12,15,16,17-octahydrocyclopenta[a]phenanthren-17-yl]-5-isopropyl-hex-5-enoic acid | 38.26 | 0.82 |
| MOL000287 | 3beta-Hydroxy-24-methylene-8-lanostene-21-oic acid | 38.7 | 0.81 |
| MOL000289 | pachymic acid | 33.63 | 0.81 |
| MOL000290 | Poricoic acid A | 30.61 | 0.76 |
| MOL000291 | Poricoic acid B | 30.52 | 0.75 |
| MOL000292 | poricoic acid C | 38.15 | 0.75 |
| MOL000296 | hederagenin | 36.91 | 0.75 |
| MOL000300 | dehydroeburicoic acid | 44.17 | 0.83 |

| **Ingredients of Banxia.** | |  |  | |
| --- | --- | --- | --- | --- |
| **Mol ID** | **Molecule name** | **OB(%)** | **DL** |  |
| MOL001755 | 24-Ethylcholest-4-en-3-one | 36.08 | 0.76 |  |
| MOL002670 | Cavidine | 35.64 | 0.81 |  |
| MOL002714 | baicalein | 33.52 | 0.21 |  |
| MOL002776 | Baicalin | 40.12 | 0.75 |  |
| MOL000358 | beta-sitosterol | 36.91 | 0.75 |  |
| MOL000449 | Stigmasterol | 43.83 | 0.76 |  |
| MOL005030 | gondoic acid | 30.7 | 0.2 |  |
| MOL000519 | coniferin | 31.11 | 0.32 |  |
| MOL006936 | 10,13-eicosadienoic | 39.99 | 0.2 |  |
| MOL006937 | 12,13-epoxy-9-hydroxynonadeca-7,10-dienoic acid | 42.15 | 0.24 |  |
| MOL006957 | (3S,6S)-3-(benzyl)-6-(4-hydroxybenzyl)piperazine-2,5-quinone | 46.89 | 0.27 |  |
| MOL003578 | Cycloartenol | 38.69 | 0.78 |  |
| MOL006967 | beta-D-Ribofuranoside, xanthine-9 | 44.72 | 0.21 |  |

| **Ingredients of Chenpi.** | |  |  |
| --- | --- | --- | --- |
| **Mol ID** | **Molecule name** | **OB(%)** | **DL** |
| MOL000359 | sitosterol | 36.91 | 0.75 |
| MOL004328 | naringenin | 59.29 | 0.21 |
| MOL005100 | 5,7-dihydroxy-2-(3-hydroxy-4-methoxyphenyl)chroman-4-one | 47.74 | 0.27 |
| MOL005815 | Citromitin | 86.9 | 0.51 |
| MOL005828 | nobiletin | 61.67 | 0.52 |

| **Ingredients of Qianhu.** | |  |  |
| --- | --- | --- | --- |
| **Mol ID** | **Molecule name** | **OB(%)** | **DL** |
| MOL013095 | Sporidesmin | 58.31 | 0.76 |
| MOL000358 | beta-sitosterol | 36.91 | 0.75 |
| MOL000359 | sitosterol | 36.91 | 0.75 |
| MOL013100 | (2S)-2-(1-hydroxy-1-methyl-ethyl)-9-[(2S,3R,4R,5S,6R)-3,4,5-trihydroxy-6-methylol-tetrahydropyran-2-yl]oxy-2,3-dihydrofuro[3,2-g]chromen-7-one | 45.33 | 0.73 |
| MOL013093 | rubricauloside | 58.36 | 0.71 |
| MOL004792 | nodakenin | 57.12 | 0.69 |
| MOL013078 | praeruptorin E | 51.22 | 0.66 |
| MOL004653 | (+)-Anomalin | 46.06 | 0.66 |
| MOL013081 | Decussine | 39.83 | 0.65 |
| MOL013079 | dl-praeruptorin a | 46.46 | 0.53 |
| MOL013087 | Peucedanocoumarin II | 63.48 | 0.53 |
| MOL013103 | 532-16-1 | 46.57 | 0.44 |
| MOL007154 | tanshinone iia | 49.89 | 0.4 |
| MOL013077 | Decursin | 39.27 | 0.38 |
| MOL013098 | [(9R)-8,8-dimethyl-2-oxo-9,10-dihydropyrano[6,5-h]chromen-9-yl](Z)-2-methylbut-2-enoate | 87.48 | 0.37 |
| MOL013083 | Skimmin (8CI) | 38.35 | 0.32 |
| MOL002644 | Phellopterin | 40.19 | 0.28 |
| MOL000098 | quercetin | 46.43 | 0.28 |
| MOL005100 | 5,7-dihydroxy-2-(3-hydroxy-4-methoxyphenyl)chroman-4-one | 47.74 | 0.27 |
| MOL001942 | isoimperatorin | 45.46 | 0.23 |
| MOL001941 | Ammidin | 34.55 | 0.22 |
| MOL013094 | 8-[(2R)-2,3-dihydroxy-3-methyl-butyl]-5,7-dimethoxy-coumarin | 48.57 | 0.21 |
| MOL013101 | rutarin_qt | 70.1 | 0.2 |
| MOL013076 | (8S,9R)-9-hydroxy-8-(2-hydroxypropan-2-yl)-8,9-dihydrofuro[2,3-h]chromen-2-one | 37.3 | 0.2 |

| **Ingredients of Danpi.** | |  |  |
| --- | --- | --- | --- |
| **Mol ID** | **Molecule name** | **OB(%)** | **DL** |
| MOL001925 | paeoniflorin_qt | 68.18 | 0.4 |
| MOL000211 | Mairin | 55.38 | 0.78 |
| MOL000359 | sitosterol | 36.91 | 0.75 |
| MOL000422 | kaempferol | 41.88 | 0.24 |
| MOL000492 | (+)-catechin | 54.83 | 0.24 |
| MOL007003 | benzoyl paeoniflorin | 31.14 | 0.54 |
| MOL007369 | 4-O-methylpaeoniflorin_qt | 67.24 | 0.43 |
| MOL007374 | 5-[[5-(4-methoxyphenyl)-2-furyl]methylene]barbituric acid | 43.44 | 0.3 |
| MOL007382 | mudanpioside-h_qt 2 | 42.36 | 0.37 |
| MOL007384 | paeonidanin_qt | 65.31 | 0.35 |
| MOL000098 | quercetin | 46.43 | 0.28 |

| **Ingredients of Gancao.** | |  |  |
| --- | --- | --- | --- |
| **Mol ID** | **Molecule name** | **OB(%)** | **DL** |
| MOL001484 | Inermine | 75.18 | 0.54 |
| MOL001792 | DFV | 32.76 | 0.18 |
| MOL000211 | Mairin | 55.38 | 0.78 |
| MOL002311 | Glycyrol | 90.78 | 0.67 |
| MOL000239 | Jaranol | 50.83 | 0.29 |
| MOL002565 | Medicarpin | 49.22 | 0.34 |
| MOL000354 | isorhamnetin | 49.6 | 0.31 |
| MOL000359 | sitosterol | 36.91 | 0.75 |
| MOL003656 | Lupiwighteone | 51.64 | 0.37 |
| MOL003896 | 7-Methoxy-2-methyl isoflavone | 42.56 | 0.2 |
| MOL000392 | formononetin | 69.67 | 0.21 |
| MOL000417 | Calycosin | 47.75 | 0.24 |
| MOL000422 | kaempferol | 41.88 | 0.24 |
| MOL004328 | naringenin | 59.29 | 0.21 |
| MOL004805 | (2S)-2-[4-hydroxy-3-(3-methylbut-2-enyl)phenyl]-8,8-dimethyl-2,3-dihydropyrano[2,3-f]chromen-4-one | 31.79 | 0.72 |
| MOL004806 | euchrenone | 30.29 | 0.57 |
| MOL004808 | glyasperin B | 65.22 | 0.44 |
| MOL004810 | glyasperin F | 75.84 | 0.54 |
| MOL004811 | Glyasperin C | 45.56 | 0.4 |
| MOL004814 | Isotrifoliol | 31.94 | 0.42 |
| MOL004815 | (E)-1-(2,4-dihydroxyphenyl)-3-(2,2-dimethylchromen-6-yl)prop-2-en-1-one | 39.62 | 0.35 |
| MOL004820 | kanzonols W | 50.48 | 0.52 |
| MOL004824 | (2S)-6-(2,4-dihydroxyphenyl)-2-(2-hydroxypropan-2-yl)-4-methoxy-2,3-dihydrofuro[3,2-g]chromen-7-one | 60.25 | 0.63 |
| MOL004827 | Semilicoisoflavone B | 48.78 | 0.55 |
| MOL004828 | Glepidotin A | 44.72 | 0.35 |
| MOL004829 | Glepidotin B | 64.46 | 0.34 |
| MOL004833 | Phaseolinisoflavan | 32.01 | 0.45 |
| MOL004835 | Glypallichalcone | 61.6 | 0.19 |
| MOL004838 | 8-(6-hydroxy-2-benzofuranyl)-2,2-dimethyl-5-chromenol | 58.44 | 0.38 |
| MOL004841 | Licochalcone B | 76.76 | 0.19 |
| MOL004848 | licochalcone G | 49.25 | 0.32 |
| MOL004849 | 3-(2,4-dihydroxyphenyl)-8-(1,1-dimethylprop-2-enyl)-7-hydroxy-5-methoxy-coumarin | 59.62 | 0.43 |
| MOL004855 | Licoricone | 63.58 | 0.47 |
| MOL004856 | Gancaonin A | 51.08 | 0.4 |
| MOL004857 | Gancaonin B | 48.79 | 0.45 |
| MOL004860 | licorice glycoside E | 32.89 | 0.27 |
| MOL004863 | 3-(3,4-dihydroxyphenyl)-5,7-dihydroxy-8-(3-methylbut-2-enyl)chromone | 66.37 | 0.41 |
| MOL004864 | 5,7-dihydroxy-3-(4-methoxyphenyl)-8-(3-methylbut-2-enyl)chromone | 30.49 | 0.41 |
| MOL004866 | 2-(3,4-dihydroxyphenyl)-5,7-dihydroxy-6-(3-methylbut-2-enyl)chromone | 44.15 | 0.41 |
| MOL004879 | Glycyrin | 52.61 | 0.47 |
| MOL004882 | Licocoumarone | 33.21 | 0.36 |
| MOL004883 | Licoisoflavone | 41.61 | 0.42 |
| MOL004884 | Licoisoflavone B | 38.93 | 0.55 |
| MOL004885 | licoisoflavanone | 52.47 | 0.54 |
| MOL004891 | shinpterocarpin | 80.3 | 0.73 |
| MOL004898 | (E)-3-[3,4-dihydroxy-5-(3-methylbut-2-enyl)phenyl]-1-(2,4-dihydroxyphenyl)prop-2-en-1-one | 46.27 | 0.31 |
| MOL004903 | liquiritin | 65.69 | 0.74 |
| MOL004904 | licopyranocoumarin | 80.36 | 0.65 |
| MOL004905 | 3,22-Dihydroxy-11-oxo-delta(12)-oleanene-27-alpha-methoxyca | 34.32 | 0.55 |
| MOL004907 | Glyzaglabrin | 61.07 | 0.35 |
| MOL004908 | Glabridin | 53.25 | 0.47 |
| MOL004910 | Glabranin | 52.9 | 0.31 |
| MOL004911 | Glabrene | 46.27 | 0.44 |
| MOL004912 | Glabrone | 52.51 | 0.5 |
| MOL004913 | 1,3-dihydroxy-9-methoxy-6-benzofurano[3,2-c]chromenone | 48.14 | 0.43 |
| MOL004914 | 1,3-dihydroxy-8,9-dimethoxy-6-benzofurano[3,2-c]chromenone | 62.9 | 0.53 |
| MOL004915 | Eurycarpin A | 43.28 | 0.37 |
| MOL004917 | glycyroside | 37.25 | 0.79 |
| MOL004924 | (-)-Medicocarpin | 40.99 | 0.95 |
| MOL004935 | Sigmoidin-B | 34.88 | 0.41 |
| MOL004941 | (2R)-7-hydroxy-2-(4-hydroxyphenyl)chroman-4-one | 71.12 | 0.18 |
| MOL004945 | (2S)-7-hydroxy-2-(4-hydroxyphenyl)-8-(3-methylbut-2-enyl)chroman-4-one | 36.57 | 0.32 |
| MOL004948 | Isoglycyrol | 44.7 | 0.84 |
| MOL004949 | Isolicoflavonol | 45.17 | 0.42 |
| MOL004957 | HMO | 38.37 | 0.21 |
| MOL004959 | 1-Methoxyphaseollidin | 69.98 | 0.64 |
| MOL004961 | Quercetin der. | 46.45 | 0.33 |
| MOL004966 | 3'-Hydroxy-4'-O-Methylglabridin | 43.71 | 0.57 |
| MOL000497 | licochalcone a | 40.79 | 0.29 |
| MOL004974 | 3'-Methoxyglabridin | 46.16 | 0.57 |
| MOL004978 | 2-[(3R)-8,8-dimethyl-3,4-dihydro-2H-pyrano[6,5-f]chromen-3-yl]-5-methoxyphenol | 36.21 | 0.52 |
| MOL004980 | Inflacoumarin A | 39.71 | 0.33 |
| MOL004985 | icos-5-enoic acid | 30.7 | 0.2 |
| MOL004988 | Kanzonol F | 32.47 | 0.89 |
| MOL004989 | 6-prenylated eriodictyol | 39.22 | 0.41 |
| MOL004990 | 7,2',4'-trihydroxy－5-methoxy-3－arylcoumarin | 83.71 | 0.27 |
| MOL004991 | 7-Acetoxy-2-methylisoflavone | 38.92 | 0.26 |
| MOL004993 | 8-prenylated eriodictyol | 53.79 | 0.4 |
| MOL004996 | gadelaidic acid | 30.7 | 0.2 |
| MOL000500 | Vestitol | 74.66 | 0.21 |
| MOL005000 | Gancaonin G | 60.44 | 0.39 |
| MOL005001 | Gancaonin H | 50.1 | 0.78 |
| MOL005003 | Licoagrocarpin | 58.81 | 0.58 |
| MOL005007 | Glyasperins M | 72.67 | 0.59 |
| MOL005008 | Glycyrrhiza flavonol A | 41.28 | 0.6 |
| MOL005012 | Licoagroisoflavone | 57.28 | 0.49 |
| MOL005013 | 18α-hydroxyglycyrrhetic acid | 41.16 | 0.71 |
| MOL005016 | Odoratin | 49.95 | 0.3 |
| MOL005017 | Phaseol | 78.77 | 0.58 |
| MOL005018 | Xambioona | 54.85 | 0.87 |
| MOL005020 | dehydroglyasperins C | 53.82 | 0.37 |
| MOL000098 | quercetin | 46.43 | 0.28 |
