# Supplementary material for: Network Pharmacology and Experimental Verification Revealed the Mechanism of Yiqi Jianpi Recipe on Chronic Obstructive Pulmonary Disease
Source: Evid Based Complement Alternat Med. 2022 Sep 7;2022:8823231. doi: 10.1155/2022/8823231 (PMC9473879; doi:10.1155/2022/8823231)
Supplement: Supplementary Materials — Supplementary Table 1: information of collected YQJPR active ingredients. Supplementary Table 2: results of Venn diagram. Supplementary Table 3: data of KEGG enrichment analysis. [file 8823231.f1.zip › Supplementary Table 2.docx]

**Supplementary Table 2: results of Venn Diagram.**

| **4832 genes included exclusively in "COPD"** | | |  |  | **35 genes included exclusively in "YQJPR"** |  | **225 intersection genes in "COPD" and "YQJPR"** |
| --- | --- | --- | --- | --- | --- | --- | --- |
| DDTL | GTF2IRD1 | RAD23B | LPL |  | HTR |  | ATP5F1B |
| MAP6D1 | BBS4 | SFN | RARB |  | CHRM4 |  | PLB1 |
| FAM149A | FANCM | GMNN | GNAS |  | CALM1 |  | NUF2 |
| LNP1 | TREM1 | TRPC4 | UCHL1 |  | PDE10A |  | NOX5 |
| ZNF780A | ERF | IGFALS | PDGFB |  | TDRD7 |  | AHSA1 |
| ZNF311 | CLN5 | SEC24D | CDH1 |  | IGHG1 |  | CTRB1 |
| FAM219B | ICOS | GJA4 | MME |  | MAOA |  | CXCL2 |
| IGLL5 | BBS2 | NFIA | CDKN2A |  | MTAP |  | SLPI |
| RUFY4 | KIR3DL1 | HSP90B1 | BRCA1 |  | BAD |  | EIF6 |
| TEPP | STX3 | CKB | ADA |  | MTTP |  | FABP5 |
| C6orf136 | NR2E3 | SUMO1 | MERTK |  | C1QB |  | PTGES |
| OR12D3 | TNFRSF8 | ANGPTL4 | HDAC4 |  | GOT1 |  | CXCL11 |
| H4C2 | RRAS | C1QBP | NT5E |  | ABAT |  | GSTM2 |
| RASSF10 | LDB3 | CASQ1 | HDAC6 |  | SOAT1 |  | FOSL2 |
| OTOL1 | GTF2I | SLC20A1 | DDR2 |  | PKIA |  | DIO1 |
| TSPO2 | KMT2D | DCT | NFKB2 |  | IL10RB |  | HAS2 |
| LY6G5B | SEC61A1 | TRPM8 | AXL |  | TOP2A |  | CLDN4 |
| CNMD | CHRNG | GPR37 | NTRK3 |  | INSRR |  | ADH1C |
| TBC1D32 | WRAP53 | GFM1 | GABPA |  | TUBB1 |  | GSTM1 |
| POLR1H | POGZ | SRD5A1 | GTF2B |  | PPP3CA |  | AKR1C1 |
| CFAP43 | NFU1 | ADAMTS5 | DHX9 |  | RUNX1T1 |  | PARP4 |
| DYNLT2B | CCK | SLC25A3 | ATP1A1 |  | PTGER3 |  | ELK1 |
| RO60 | NOX4 | ARAF | WNT5A |  | IL8RA |  | E2F1 |
| CYS1 | SERPINE2 | CFL1 | GCK |  | SULT1E1 |  | PSMD3 |
| TENT5A | RPS27A | S1PR1 | GRIN1 |  | DCAF5 |  | MGAM |
| DYNC2I1 | POLG2 | CA9 | ITGB1 |  | HSF1 |  | MAP2 |
| DEFB118 | CCNO | ALPI | CTSK |  | E2F2 |  | OLR1 |
| HIGD1B | CRB2 | ADARB1 | CACNA1C |  | ACPP |  | CD163 |
| AMER3 | CLCA1 | CANT1 | NR5A1 |  | PCOLCE |  | MAOB |
| ZBED5 | GP1BB | PEBP1 | HNF4A |  | NPEPPS |  | SELE |
| ANKRD37 | AFF4 | BRD2 | PTH1R |  | GRIA2 |  | SOAT2 |
| TMEM26 | NIPBL | TNKS | PCSK9 |  | ADH1B |  | FOSL1 |
| LRRC49 | HAVCR1 | FDFT1 | ALDH2 |  | LYZD1 |  | HSD3B1 |
| VWA8 | GCG | CSTA | DNMT3B |  | ND6 |  | ALOX12 |
| CNPY1 | GDF15 | KLF5 | CDC42 |  | BACE1 |  | APOD |
| OR12D2 | EDAR | CHST6 | BMPR1B |  |  |  | RASSF1 |
| EFCAB5 | DTNBP1 | FBLN1 | BMPR1A |  |  |  | CCNA2 |
| C4orf33 | MATR3 | SIRT6 | VIM |  |  |  | DUOX2 |
| BTBD16 | LMOD1 | PDE7A | LRP1B |  |  |  | CXCL10 |
| PLAC9 | BACH2 | TK1 | TEP1 |  |  |  | TIMP1 |
| FBXL22 | ZMPSTE24 | SLC22A3 | DIO3 |  |  |  | IL1A |
| CFAP58 | ATP4A | PTPN3 | RAB38 |  |  |  | CHRM5 |
| WDR64 | SAMHD1 | KCNN3 | CLSTN2 |  |  |  | ADCY2 |
| KYAT3 | PEX6 | PSMA7 | MMRN1 |  |  |  | NCOA1 |
| C1orf162 | CXCL1 | TACSTD2 | PKNOX1 |  |  |  | OPRD1 |
| IQCD | PALB2 | NNMT | TAOK2 |  |  |  | NCOA2 |
| LEKR1 | NEK8 | CDC6 | FPR3 |  |  |  | NR1I3 |
| CFAP20 | GJC2 | PAX1 | ANGPTL6 |  |  |  | DRD1 |
| SPINK13 | DSTYK | CYP2J2 | MTHFD2 |  |  |  | CDK1 |
| MORN1 | GNPTAB | POLR1D | HLX |  |  |  | TNFSF15 |
| BPIFA2 | S100A9 | CCKBR | HOXB2 |  |  |  | F3 |
| DNAH14 | NDE1 | UMPS | CDH7 |  |  |  | THBD |
| TMEM80 | SAA1 | FGF14 | CLDN6 |  |  |  | IL2 |
| ZSCAN31 | IFNB1 | RGS9 | SIGIRR |  |  |  | IL4 |
| ZSWIM8 | SMN2 | NDUFA12 | SCUBE2 |  |  |  | ADRA1D |
| CCDC38 | S100A8 | KISS1R | HOXA11 |  |  |  | ADRA2C |
| C5orf46 | LPA | AKAP9 | RBM4 |  |  |  | AKR1C3 |
| MRPL58 | HBG2 | SLC8A1 | E2F5 |  |  |  | CHRM1 |
| ZSCAN9 | ZFPM2 | PKM | MCF2 |  |  |  | HSD3B2 |
| TMEM169 | TINF2 | P2RX4 | DYNLL1 |  |  |  | CCNB1 |
| HECTD4 | FGF7 | PRDM1 | NMB |  |  |  | NR1I2 |
| MRM2 | TBX18 | NONO | MED1 |  |  |  | SLC2A4 |
| CFAP70 | GM2A | TNNI2 | ITPK1 |  |  |  | LTA4H |
| ARHGEF38 | DNAI1 | TBX3 | SLC17A6 |  |  |  | RASA1 |
| ESX1 | HLA-DPA1 | CDH13 | ELMO1 |  |  |  | BIRC5 |
| NME9 | STRA6 | PPP2R2B | NLRX1 |  |  |  | HMGCR |
| CFAP44 | TAC1 | FOXP2 | MYOM1 |  |  |  | HSPA5 |
| H2AC20 | IQCB1 | BIRC2 | SLC4A3 |  |  |  | PPARA |
| FREM3 | TREX1 | ADIPOR1 | BACH1 |  |  |  | CYP1A2 |
| RABL2B | AHI1 | TP53BP1 | RAB35 |  |  |  | MCL1 |
| H4C1 | IFNA2 | RPL35 | HUS1 |  |  |  | BCL2L1 |
| ARL13A | HHIP | BID | PCSK5 |  |  |  | IGFBP3 |
| VWA7 | CEACAM6 | HPSE | CLDN7 |  |  |  | ACHE |
| IFT22 | CD80 | EGLN2 | SLC10A2 |  |  |  | PRSS1 |
| OR5V1 | RPGR | IL1RAP | PHEX |  |  |  | VCAM1 |
| CSH2 | SST | TMPRSS6 | FANCI |  |  |  | SREBF1 |
| SLC45A1 | ALG9 | TRIO | SHBG |  |  |  | SPP1 |
| CGB7 | HSPA4 | CTSS | ANKH |  |  |  | APOB |
| RSPH6A | NPHS2 | PLTP | PIGN |  |  |  | CRP |
| TICRR | LTBP4 | CASQ2 | PRKD1 |  |  |  | CHRNA2 |
| UQCC3 | IL17F | CHRNB1 | MYB |  |  |  | HK2 |
| PATJ | LTA | FA2H | SLC12A6 |  |  |  | ADRA1B |
| CRACR2B | ALMS1 | PNMT | RFX3 |  |  |  | CES1 |
| CTAG1B | IGHMBP2 | WASF1 | UQCRQ |  |  |  | ADRA2A |
| SEPTIN5 | PMP22 | EHMT2 | SIX2 |  |  |  | RXRB |
| SELENOP | TBX4 | XRCC6 | PCTP |  |  |  | ADRA1A |
| SGO2 | SETBP1 | TUBB4A | CYFIP1 |  |  |  | PPARD |
| UTP4 | IL17A | LTB4R | PPP1R15A |  |  |  | NFATC1 |
| HYKK | CAMK1 | LIG3 | NUBP1 |  |  |  | AKR1B1 |
| PSORS1C2 | ARHGAP5 | RELB | SMPD3 |  |  |  | HTR3A |
| CGB3 | RGS10 | ISCU | RECQL |  |  |  | PRKCB |
| TMEM240 | KAT7 | ARG2 | HOXB7 |  |  |  | RB1 |
| DNAH6 | CYP2S1 | NDUFA10 | GCA |  |  |  | CASP9 |
| BBIP1 | CHRNA6 | PDE4B | BTN3A1 |  |  |  | PTGS1 |
| H4-16 | KCNH7 | ROBO1 | EHD1 |  |  |  | RUNX2 |
| RAB7B | BRS3 | TAT | TOP3B |  |  |  | NR3C2 |
| CCN1 | DUSP10 | PAFAH1B1 | RARRES1 |  |  |  | IRF1 |
| NSMCE3 | CDH6 | ERG | RPH3A |  |  |  | TYR |
| KIFBP | TLE3 | TFPI | SGMS1 |  |  |  | BAX |
| DYNC2I2 | PTPN7 | ATP6V1B1 | SERPINA10 |  |  |  | COL3A1 |
| WASHC5 | SLC16A7 | ATP6V0A2 | PCMT1 |  |  |  | PYGM |
| C11orf65 | ANXA3 | RPL18 | HOXA3 |  |  |  | CD40LG |
| MT-ATP8 | HNRNPD | FKBP5 | CCNC |  |  |  | HIF1A |
| DNAAF11 | GPAM | CDC45 | PABPC4 |  |  |  | PON1 |
| CFAP298 | DUSP4 | CDKN2C | SLC5A11 |  |  |  | EDN3 |
| DNAAF4 | GNG2 | PPP2R5D | NKX3-1 |  |  |  | CAV1 |
| C12orf60 | PRDX4 | SLC16A2 | KIFC1 |  |  |  | NCF1 |
| NT5C3B | CDK19 | SLC22A2 | F2RL2 |  |  |  | IL1B |
| MAGEA9 | DDIT4 | RAB5A | TAGLN2 |  |  |  | GABRA1 |
| ANKRD7 | CCNA1 | ANK1 | HPGDS |  |  |  | SLC6A2 |
| ZNF319 | RBL2 | RAPGEF3 | HSD17B8 |  |  |  | ODC1 |
| GLIPR1L2 | HGFAC | DISC1 | EGR3 |  |  |  | AHR |
| BTBD7 | GRIK5 | KRT17 | MYBL1 |  |  |  | NQO1 |
| TM6SF1 | MAPKAP1 | TCF3 | ADGRE5 |  |  |  | HSP90AA1 |
| HMGN4 | NISCH | SPTA1 | CAPZB |  |  |  | IGF2 |
| TMEM106C | TCP1 | SLC10A1 | MPP1 |  |  |  | XIAP |
| CCT8L2 | CA14 | GPC4 | INCENP |  |  |  | CDKN1A |
| TIMM21 | INHBB | HNRNPK | MYH1 |  |  |  | JUN |
| RBM33 | HTR5A | CHD2 | GALNT12 |  |  |  | CYP1A1 |
| OR2B2 | WASF2 | KLF4 | AZU1 |  |  |  | CYCS |
| SPHKAP | ACTR2 | EYA4 | MID2 |  |  |  | TP63 |
| MAP3K19 | SLC4A5 | MTHFD1 | CLTCL1 |  |  |  | SLC6A4 |
| H3-4 | NFYC | AOC3 | TUBA1A |  |  |  | VEGFA |
| ZSCAN16 | FGFRL1 | XYLT2 | VCAN |  |  |  | CCL2 |
| TMEM170A | HSF2 | ARL3 | LEF1 |  |  |  | CHRM2 |
| VSIR | SEMA3F | NDUFA1 | RAB27A |  |  |  | ACACA |
| GPN2 | PLXNA2 | GSTO1 | DHFR |  |  |  | EGLN1 |
| DMBX1 | SH3GL2 | SOCS2 | PRDX1 |  |  |  | FASN |
| ASCL3 | RGS4 | ALOX15 | USP8 |  |  |  | CHEK1 |
| INO80E | CD74 | BLVRA | FANCA |  |  |  | RXRA |
| CCDC61 | RANBP1 | FEN1 | PGM1 |  |  |  | CALCR |
| ZSCAN26 | RXFP2 | PABPN1 | COMP |  |  |  | CASP7 |
| WBP1L | SEC31A | ALAS2 | YY1 |  |  |  | TOP1 |
| ABHD12B | QKI | ADAMTS4 | ASS1 |  |  |  | PLA2G4A |
| PRR3 | TFB1M | B3GAT1 | SLC12A1 |  |  |  | ADRB1 |
| RMDN1 | SEMA7A | GFRA1 | ROR2 |  |  |  | ABCC1 |
| ZSCAN12 | PIK3R3 | STAG2 | ATP1A3 |  |  |  | ESR2 |
| CYYR1 | VASP | SATB1 | ALDOA |  |  |  | OPRM1 |
| CAPN14 | RHCG | NME1 | CYP2A6 |  |  |  | PARP1 |
| DNAH12 | PDE9A | SALL4 | CCND2 |  |  |  | PGR |
| ZKSCAN8 | TRAP1 | TBL1XR1 | CDK8 |  |  |  | GSK3B |
| RSPH14 | ITGA11 | KCNJ8 | SPTAN1 |  |  |  | UGT1A1 |
| LEPROT | USP11 | HTR4 | FLI1 |  |  |  | MAPK8 |
| SUPT7L | CCNK | IRF6 | P2RY12 |  |  |  | NPM1 |
| CFAP69 | NRF1 | CCNH | IL6ST |  |  |  | CYP19A1 |
| OR10AD1 | CDA | ENPP2 | NRXN1 |  |  |  | MAPK3 |
| COMMD10 | CBR3 | UNC13D | BMP1 |  |  |  | F7 |
| SPATA9 | TMLHE | APTX | LRP6 |  |  |  | CREB1 |
| H3C1 | NMT1 | TNXB | LRP2 |  |  |  | NFE2L2 |
| SH2D4B | CPD | SLC26A2 | CASP10 |  |  |  | GSR |
| ELOB | SP3 | SLC9A3R1 | PIK3CG |  |  |  | CYP1B1 |
| ZBTB9 | GJA3 | RPSA | MYL2 |  |  |  | CHRM3 |
| LIPM | EIF2AK1 | SGCD | EPCAM |  |  |  | MMP8 |
| ELOC | SERPINB8 | ADRB3 | KCNJ11 |  |  |  | ECE1 |
| ZAN | SLIT3 | DLK1 | MSH2 |  |  |  | PLAT |
| JAKMIP3 | AKR1B10 | RAP1A | RHO |  |  |  | CASP3 |
| CFAP52 | LCP2 | TFAP2B | ITGA4 |  |  |  | ALOX5 |
| CCDC97 | FOXA1 | POLA1 | MLH1 |  |  |  | NOS2 |
| KDF1 | POLL | A2M | SIRT1 |  |  |  | PTGS2 |
| RMDN2 | MLF1 | ERCC5 | ITGA3 |  |  |  | ADRB2 |
| KAZN | RUVBL1 | IRS2 | CETP |  |  |  | IL6 |
| IFT46 | SLC23A2 | GAD2 | BRIP1 |  |  |  | MAPK10 |
| WAPL | DKK2 | INPP5D | NF2 |  |  |  | POR |
| FBF1 | MYBL2 | CMA1 | MMP7 |  |  |  | ABCG2 |
| PRRC2A | SLC23A1 | C5AR1 | GSN |  |  |  | PRKCA |
| SEPTIN2 | CYP3A7 | CDC73 | MVK |  |  |  | CA2 |
| H2BC21 | RPN1 | AICDA | SLC4A1 |  |  |  | PCNA |
| PAGR1 | HES1 | TAPBP | RYR2 |  |  |  | DPP4 |
| CCN3 | AP1B1 | DPAGT1 | GAPDH |  |  |  | FOS |
| KBTBD13 | NFATC2 | CRHR1 | CACNA1A |  |  |  | CYP3A4 |
| ATP5F1E | SULF1 | HERC2 | ADAMTS13 |  |  |  | KCNH2 |
| OGA | SUV39H1 | GPNMB | WAS |  |  |  | SLC6A3 |
| DGCR6 | NFATC3 | GFER | IL4R |  |  |  | ITGB3 |
| AOPEP | UQCRC1 | HDC | GRN |  |  |  | NFKBIA |
| EFL1 | SKIL | GNS | PAX6 |  |  |  | RELA |
| SPPL2C | CDT1 | XBP1 | POMC |  |  |  | IFNGR1 |
| VPS37D | RYK | SYT2 | KRT18 |  |  |  | LDLR |
| BUD23 | NEUROD2 | HAND2 | GATA2 |  |  |  | GJA1 |
| MT-ND3 | GSTA1 | LTBP1 | PLA2G6 |  |  |  | NR3C1 |
| DRC1 | GRK5 | SI | TFRC |  |  |  | SCN5A |
| SELENON | MSMO1 | DVL3 | CSF2RB |  |  |  | CAT |
| GSTT1 | MTHFS | EHMT1 | SMAD2 |  |  |  | MMP3 |
| MT-ND6 | ING1 | MYL3 | GUSB |  |  |  | GSTP1 |
| MT-ND4 | ALDH3A1 | DDIT3 | CYP2D6 |  |  |  | BCL2 |
| MT-CO3 | KHDRBS1 | FCN3 | F9 |  |  |  | SERPINE1 |
| MT-CYB | TNFRSF12A | LARS2 | CD40 |  |  |  | EDNRA |
| STN1 | CD96 | ADD1 | C3 |  |  |  | ICAM1 |
| MT-ND1 | NEDD9 | NLRC4 | HEXB |  |  |  | EPHB2 |
| BARHL2 | CELSR2 | EZR | JUP |  |  |  | CDK2 |
| ARRDC1 | NUDC | RFC2 | HTR2A |  |  |  | CHUK |
| MGARP | MYL1 | SMARCE1 | ACTA1 |  |  |  | MMP13 |
| PRSS35 | GSTA4 | FUT2 | CD36 |  |  |  | CTSD |
| YPEL1 | ELOVL5 | GPC3 | TNFRSF11B |  |  |  | MAPK14 |
| SCFD2 | JAG2 | ACADVL | RUNX1 |  |  |  | MYC |
| SLC38A6 | PPP1R12A | SHC1 | NRAS |  |  |  | PLAU |
| TMED3 | CHST11 | BDKRB2 | B2M |  |  |  | CASP8 |
| ZNF546 | LILRB1 | PLN | APC |  |  |  | EGF |
| TMEM200A | PSMA2 | EFEMP1 | ABCA1 |  |  |  | MAPK1 |
| HILPDA | VDAC2 | STUB1 | PMS2 |  |  |  | HSPB1 |
| TMEM182 | RAPGEF4 | CD33 | ELANE |  |  |  | COL1A1 |
| ZNF839 | NIN | MAGI2 | MTHFR |  |  |  | CCND1 |
| ARHGEF37 | FAF1 | COL6A1 | GATA4 |  |  |  | SOD1 |
| TUBGCP5 | RAB3A | MSTN | EDN1 |  |  |  | APP |
| NSD3 | SLC4A7 | PURA | GLA |  |  |  | HMOX1 |
| CELF6 | EEA1 | RNASEL | IFNG |  |  |  | MPO |
| RAPGEFL1 | ST6GAL1 | LBP | CSNK1A1 |  |  |  | STAT3 |
| CAMTA2 | MTO1 | SATB2 | ADCY1 |  |  |  | NOS3 |
| IFNA17 | PTAFR | PSMB4 | ESRRB |  |  |  | IKBKB |
| REM1 | SLC7A6 | ALOX5AP | PRKACB |  |  |  | CDK4 |
| SRXN1 | CD82 | CFLAR | PDPK1 |  |  |  | MDM2 |
| DENND2D | PDGFC | CHRNA5 | PRKCE |  |  |  | CHEK2 |
| DTWD1 | CALD1 | HRH2 | HDAC3 |  |  |  | KDR |
| SNRNP35 | KCNMB1 | GP6 | MST1R |  |  |  | ESR1 |
| DGCR6L | RTN4R | RORC | NR1D1 |  |  |  | AR |
| B4GALNT4 | VAPA | MUTYH | ADAM9 |  |  |  | MMP2 |
| TRIM15 | PTBP1 | BTD | RDX |  |  |  | PPARG |
| MTUS2 | GIPC1 | PIGA | PIK3CB |  |  |  | AKT1 |
| HMGXB3 | CTSE | FBXW7 | PBX1 |  |  |  | STAT1 |
| TMEM51 | FLII | TAF1 | SCD |  |  |  | MMP1 |
| TTC7B | KERA | TIMP3 | MAP2K3 |  |  |  | MET |
| TIPARP | SLC1A7 | TRPM4 | PIM1 |  |  |  | ERBB3 |
| C15orf40 | PSMA3 | MYO5B | PDE3A |  |  |  | RAF1 |
| TTLL9 | CRBN | FOXO3 | YWHAE |  |  |  | EGFR |
| FUT11 | MAP3K13 | BECN1 | MASP1 |  |  |  | ERBB2 |
| ENDOU | HRH3 | SALL1 | BUB1 |  |  |  | MMP9 |
| MSL1 | SLC25A21 | ANG | MAP3K1 |  |  |  |  |
| NAF1 | FSTL1 | CS | NPR2 |  |  |  |  |
| COL28A1 | PSMB5 | DLL1 | PNLIP |  |  |  |  |
| SNRNP48 | UBE2T | POMT1 | CDH11 |  |  |  |  |
| ZCCHC10 | PSMD2 | ANXA11 | THRA |  |  |  |  |
| HTN3 | ACKR3 | HSPA1L | MARK3 |  |  |  |  |
| AGBL4 | ARRB1 | HTR1B | ADCY5 |  |  |  |  |
| MTMR11 | LETM1 | TPH1 | WNT7A |  |  |  |  |
| FUNDC1 | MACF1 | DKK1 | CACNA1B |  |  |  |  |
| ZNF141 | ARSG | ADCY10 | PCSK1 |  |  |  |  |
| GSX2 | PSMD14 | POFUT1 | PLCB1 |  |  |  |  |
| GPATCH3 | LMX1A | CLCN6 | PTPN6 |  |  |  |  |
| THAP7 | PSMA1 | HTRA1 | RAD50 |  |  |  |  |
| PIH1D2 | HYAL2 | IDO1 | ACTN1 |  |  |  |  |
| GPX6 | IGFBP4 | OGG1 | CACNA1G |  |  |  |  |
| RABL2A | SENP1 | MYO5A | TBXA2R |  |  |  |  |
| RRP15 | PROX1 | CACNA1F | CAPN1 |  |  |  |  |
| KANSL2 | BST1 | KRT19 | PIK3R2 |  |  |  |  |
| ORAI3 | AUH | APOA5 | FGG |  |  |  |  |
| DYDC2 | IFI16 | NEU1 | PTGER2 |  |  |  |  |
| ZBTB12 | NUDT1 | SERPINA6 | FTH1 |  |  |  |  |
| ROMO1 | SULT1A1 | PEPD | CYLD |  |  |  |  |
| SYNPO2L | DHPS | SNRPN | FZD6 |  |  |  |  |
| SIGLEC14 | LYVE1 | APOA2 | PRKCZ |  |  |  |  |
| TRMT61B | CRLF1 | PRMT7 | LAMB1 |  |  |  |  |
| GPANK1 | TFAP2C | SERPINF2 | HCN4 |  |  |  |  |
| TMEM219 | RHOH | ALPP | KCNJ1 |  |  |  |  |
| CLASRP | SEMA3E | TRPM7 | SMARCA2 |  |  |  |  |
| LRIF1 | DIS3L2 | ALDH18A1 | EIF4E |  |  |  |  |
| DNAJB8 | IGFBP6 | AHSG | TUBB |  |  |  |  |
| SNTG1 | MYL4 | SEC24C | AXIN2 |  |  |  |  |
| JRKL | LNX1 | APOC3 | HCK |  |  |  |  |
| IQCG | ESD | SP1 | FOXO1 |  |  |  |  |
| ZNF230 | GPD1L | HMGA2 | RORA |  |  |  |  |
| C12orf43 | SPOP | EYA1 | HDAC1 |  |  |  |  |
| FAM168A | PCSK2 | SCN1B | MEF2C |  |  |  |  |
| NRM | BAMBI | SLC2A10 | ANXA1 |  |  |  |  |
| PGA3 | GGH | WRN | IFNAR2 |  |  |  |  |
| MUC21 | PHF21A | COL11A2 | SLCO1B1 |  |  |  |  |
| CCDC57 | LTB4R2 | DGUOK | CYP11A1 |  |  |  |  |
| H1-5 | VAMP2 | GYG1 | ITGB4 |  |  |  |  |
| WHAMM | MC3R | CRYAB | PLK1 |  |  |  |  |
| LIPJ | ADAM19 | TLL1 | EXT1 |  |  |  |  |
| MTCL1 | SERPINB5 | MAN2B1 | EIF2AK3 |  |  |  |  |
| GUCY1B1 | KDM6B | SERPINF1 | CACNA1S |  |  |  |  |
| CLUH | MEIS2 | ITGAX | RARA |  |  |  |  |
| NSRP1 | EIF2S1 | PKP2 | LYN |  |  |  |  |
| RPAP3 | HS6ST1 | PEX1 | CHRNA4 |  |  |  |  |
| PRDM15 | ID2 | STK36 | ATP2A1 |  |  |  |  |
| CTRB2 | GRIP1 | EMD | PDHA1 |  |  |  |  |
| INTS12 | PPP1R1B | PLAUR | SLC12A2 |  |  |  |  |
| PGAM5 | SLC24A4 | ERAP1 | IRAK1 |  |  |  |  |
| CCL23 | ADAM8 | PNPLA2 | ACTG1 |  |  |  |  |
| ZKSCAN3 | IL1RAPL1 | LTBP2 | DMPK |  |  |  |  |
| RMI2 | HSD17B1 | CCR2 | CYP17A1 |  |  |  |  |
| DNAJC18 | S100A6 | C4B | GNAQ |  |  |  |  |
| COL6A5 | CALM3 | PMPCA | LIMK1 |  |  |  |  |
| CARS1 | CHD3 | SOS2 | EPAS1 |  |  |  |  |
| DPF3 | PSMA5 | LAMA2 | ACAN |  |  |  |  |
| TBXT | AKAP13 | TNFRSF10A | CYP2C19 |  |  |  |  |
| KMT5B | TAGLN | THBS1 | ITPR1 |  |  |  |  |
| DNTTIP2 | EXO1 | TRAF3IP2 | LRP1 |  |  |  |  |
| SCLT1 | HS2ST1 | BMP7 | CAPN3 |  |  |  |  |
| MESD | P2RX3 | MBP | PAH |  |  |  |  |
| UNC80 | GJC1 | MED12 | MSH6 |  |  |  |  |
| VEGFD | TMC6 | MKI67 | ACTB |  |  |  |  |
| ARSH | DMP1 | SMARCB1 | SMARCA4 |  |  |  |  |
| IGLON5 | CTNND2 | CIITA | TPM1 |  |  |  |  |
| ZNF469 | GAP43 | COL17A1 | ACE2 |  |  |  |  |
| TMEM260 | LHX1 | CTRC | STAT6 |  |  |  |  |
| CFAP53 | TFE3 | SMARCAL1 | PROS1 |  |  |  |  |
| TAFAZZIN | GADD45A | KMT2A | CYP2C9 |  |  |  |  |
| LARGE1 | RAB23 | PON2 | RAC1 |  |  |  |  |
| B3GLCT | ELAC2 | CYP2E1 | TRPC6 |  |  |  |  |
| IFNL3 | KDM5C | TNFSF10 | SCNN1B |  |  |  |  |
| COQ8B | CAV2 | TAP2 | GLI3 |  |  |  |  |
| STING1 | FCN2 | NPY | NR1H4 |  |  |  |  |
| TAMM41 | FANCG | CCR7 | CBS |  |  |  |  |
| ADA2 | THBS4 | BCS1L | PSMB8 |  |  |  |  |
| MT-CO2 | CENPF | ABCA7 | PDE4D |  |  |  |  |
| MT-ND5 | GPC1 | POSTN | ABCC2 |  |  |  |  |
| TWNK | HEY1 | LMX1B | IL2RB |  |  |  |  |
| MT-ATP6 | CNGB1 | CLCN1 | ARG1 |  |  |  |  |
| MT-CO1 | GZMA | LTF | TBK1 |  |  |  |  |
| HIGD2A | DLG1 | HNF1A | MYH9 |  |  |  |  |
| FOXD4 | ITGA1 | HNMT | TPO |  |  |  |  |
| ARMCX2 | DNAJB1 | CTNS | PRKCQ |  |  |  |  |
| MAGEC1 | CTTN | CDH23 | MYD88 |  |  |  |  |
| RCSD1 | LRP8 | VIP | GYS1 |  |  |  |  |
| FRY | DAB2 | FCGR3A | IL6R |  |  |  |  |
| FEV | TPP2 | TCIRG1 | IDS |  |  |  |  |
| KLHDC3 | INHA | HSPA1A | CD19 |  |  |  |  |
| PDRG1 | KEL | PSTPIP1 | BMP4 |  |  |  |  |
| ZDHHC18 | FGF4 | COL7A1 | BLM |  |  |  |  |
| FBXO33 | COX15 | NAGA | GGT1 |  |  |  |  |
| TIGD6 | DTNA | SDHC | FGA |  |  |  |  |
| CHCHD1 | SOX4 | POMGNT1 | SHH |  |  |  |  |
| CCDC69 | SH2B1 | TXN | STK11 |  |  |  |  |
| PPP4R4 | ITPKC | ADAR | NOTCH3 |  |  |  |  |
| IQCA1 | GPX2 | IREB2 | SLC25A4 |  |  |  |  |
| NUFIP1 | NDUFB8 | ARSB | GLB1 |  |  |  |  |
| HUS1B | SRD5A2 | KITLG | NTRK1 |  |  |  |  |
| CCDC86 | UBE3B | IL17RA | STAT5B |  |  |  |  |
| CCDC63 | TACR2 | SGSH | RRM2B |  |  |  |  |
| P3H4 | AMFR | PRF1 | G6PD |  |  |  |  |
| IFNA10 | RUNX3 | SAG | ENPP1 |  |  |  |  |
| AMZ1 | UGT1A4 | RAD21 | KCNN4 |  |  |  |  |
| NUDT13 | PLS3 | CHI3L1 | TF |  |  |  |  |
| QPCTL | MLH3 | TNFSF13B | LIPA |  |  |  |  |
| USP35 | FAM20C | S100B | PLG |  |  |  |  |
| CTAGE1 | SLC25A24 | OCRL | LRP5 |  |  |  |  |
| LIPT2 | SELPLG | CHGA | CPT2 |  |  |  |  |
| WDR20 | VANGL2 | CX3CR1 | COL1A2 |  |  |  |  |
| C1orf87 | CADM1 | APOH | COL4A1 |  |  |  |  |
| SLC48A1 | VARS2 | CCR4 | DNM2 |  |  |  |  |
| NUTM1 | KCNAB2 | ADM | TNNT2 |  |  |  |  |
| PCDHA13 | PIGQ | FLNC | TNNI3 |  |  |  |  |
| SPDYA | LDLRAP1 | PHKA2 | COL2A1 |  |  |  |  |
| MREG | SLC4A2 | CHRNA3 | PRKAG2 |  |  |  |  |
| MZB1 | FBXO11 | SYP | SERPINC1 |  |  |  |  |
| SLC35F3 | GALE | SCO2 | CP |  |  |  |  |
| UTP15 | CPLX1 | PRKCSH | IGF1 |  |  |  |  |
| PID1 | MOGS | SCARB2 | SMAD3 |  |  |  |  |
| FAM131B | OPCML | HMGB1 | TNFRSF1A |  |  |  |  |
| DXO | PXDN | PDE5A | MIF |  |  |  |  |
| FGFBP2 | PLA2G10 | IL12RB1 | GATA3 |  |  |  |  |
| CYLC2 | NARS2 | SYNJ1 | CD4 |  |  |  |  |
| CALN1 | MSRA | GC | APOA1 |  |  |  |  |
| C1orf127 | RPS27 | MECP2 | TTR |  |  |  |  |
| C1QTNF9 | TCN2 | GALC | VCP |  |  |  |  |
| COA3 | SREBF2 | AIRE | MUC1 |  |  |  |  |
| PELI2 | TNFRSF25 | RBP4 | WT1 |  |  |  |  |
| CROCC | SGCG | CXCL12 | TSC2 |  |  |  |  |
| SMG9 | CELSR1 | AGER | IL1RN |  |  |  |  |
| IGKC | ADIPOR2 | C4A | BRCA2 |  |  |  |  |
| COBL | MLX | HLA-G | LRRK2 |  |  |  |  |
| COL26A1 | CLCN3 | PDE4A | TRPV4 |  |  |  |  |
| H3-3A | CLCN4 | HLA-C | REN |  |  |  |  |
| ADAT3 | NR0B2 | KIF1B | EDNRB |  |  |  |  |
| FNDC5 | RPS14 | APOL1 | F2 |  |  |  |  |
| BPIFB1 | SGCA | CLEC7A | FLNA |  |  |  |  |
| IL26 | NAA10 | COL4A3 | SMPD1 |  |  |  |  |
| PTPA | KLF6 | IL10RA | NF1 |  |  |  |  |
| PIGY | GAS1 | IL23R | VWF |  |  |  |  |
| C14orf39 | ADAMTS10 | CXCR3 | DSP |  |  |  |  |
| GREB1L | CHIA | LAMP2 | ACVRL1 |  |  |  |  |
| SEPTIN9 | TRPM3 | GUCY2C | GAA |  |  |  |  |
| BBS12 | AIMP1 | ADIPOQ | ALB |  |  |  |  |
| GTF2IRD2 | TNFRSF6B | FIG4 | LMNA |  |  |  |  |
| SCT | HLA-E | DYNC1H1 | GBA |  |  |  |  |
| GAS2L2 | EDA | AGL | ABCA3 |  |  |  |  |
| MT-ND2 | PCDH19 | COL4A4 | SERPINA1 |  |  |  |  |
| SGO1 | COX6B1 | COL4A5 | HSD11B1 |  |  |  |  |
| GNL3L | ROBO2 | PARK7 | GABRB3 |  |  |  |  |
| KRT76 | CORO1A | TET2 | ADK |  |  |  |  |
| MAPK8IP2 | XRCC4 | IL5 | ROCK1 |  |  |  |  |
| ENOSF1 | ITIH4 | CSF2RA | PRKDC |  |  |  |  |
| ZNF496 | PLA2G2D | SERPINA3 | PPIB |  |  |  |  |
| CPO | PSMD12 | CCR6 | SLC9A1 |  |  |  |  |
| TMTC4 | PROCR | HLA-DPB1 | BUB1B |  |  |  |  |
| RAB20 | ATG5 | NKX2-5 | CTH |  |  |  |  |
| POLR3G | CNP | MPZ | CACNA1H |  |  |  |  |
| ANXA10 | KAT6A | ELN | TACR3 |  |  |  |  |
| SYNE3 | ERLIN1 | EIF2AK4 | AHCY |  |  |  |  |
| ZFYVE28 | SOX17 | CTLA4 | ACVR2B |  |  |  |  |
| SYT9 | CRTAP | SFTPB | NR2F2 |  |  |  |  |
| ZNF330 | RPL27 | FRK | GLUD1 |  |  |  |  |
| SNX8 | CYP7A1 | ADCYAP1R1 | ANPEP |  |  |  |  |
| RBP7 | CSRP3 | MAPK13 | ITGA6 |  |  |  |  |
| SEMG2 | GPR35 | PRKAB2 | FSHR |  |  |  |  |
| ZNF536 | TNFRSF9 | ACACB | AURKA |  |  |  |  |
| ZNF607 | ATG7 | PTPRA | PTPN1 |  |  |  |  |
| MTERF1 | TSPO | P2RY1 | TNC |  |  |  |  |
| ANKRD44 | ATXN10 | CASP14 | KCNMA1 |  |  |  |  |
| ALG10 | NDN | GABRB1 | MGMT |  |  |  |  |
| OXCT2 | ALG1 | MEF2D | HK1 |  |  |  |  |
| WSCD1 | UGCG | USP15 | GAD1 |  |  |  |  |
| NREP | RPS17 | PAK2 | BLK |  |  |  |  |
| ERO1A | CANX | DOCK1 | PROC |  |  |  |  |
| KRT34 | CD9 | NME2 | TNFRSF10B |  |  |  |  |
| MOK | LGALS1 | ENPEP | FN1 |  |  |  |  |
| SPTBN5 | COL9A1 | NUMB | AGT |  |  |  |  |
| SERTAD1 | ABCB7 | OXTR | IL2RA |  |  |  |  |
| FBXL7 | GSTO2 | DVL2 | JAG1 |  |  |  |  |
| ATRNL1 | DAXX | RPS6KA2 | FAS |  |  |  |  |
| MYO16 | PROKR2 | MAP2K7 | SMAD4 |  |  |  |  |
| SPATS2L | IGFBP2 | PRDX6 | MAPT |  |  |  |  |
| ASB8 | CHST3 | ARRB2 | NOTCH1 |  |  |  |  |
| CTTNBP2 | KRT13 | BTRC | KCNK3 |  |  |  |  |
| TTLL3 | POLR3A | PAX9 | SNCA |  |  |  |  |
| AKNA | HAX1 | RPA1 | KRAS |  |  |  |  |
| USP54 | SLC22A1 | PDK1 | APOE |  |  |  |  |
| ZNF18 | ACP1 | UQCRC2 | BMPR2 |  |  |  |  |
| ASPRV1 | TRIP11 | RALA | TBXAS1 |  |  |  |  |
| SPPL3 | CPOX | GPT2 | PRKAA2 |  |  |  |  |
| TM9SF4 | NUP98 | PRKG2 | FGFR4 |  |  |  |  |
| ENKUR | BMI1 | ETV1 | GRIA3 |  |  |  |  |
| GLYATL1 | SPI1 | TOP2B | GRM1 |  |  |  |  |
| ERP29 | PYY | ATP2B2 | PRKACA |  |  |  |  |
| KIF27 | TSFM | KDM4B | IDH2 |  |  |  |  |
| RASEF | SMAD1 | PSPH | MAP3K7 |  |  |  |  |
| TRIM10 | SIX1 | HTR1D | CSF1R |  |  |  |  |
| SHKBP1 | FOXE1 | EGLN3 | SPARC |  |  |  |  |
| ALDH16A1 | LMNB2 | EXTL3 | MMP14 |  |  |  |  |
| TEKT4 | TNNT1 | TNFRSF21 | IDH1 |  |  |  |  |
| PTCD1 | GPX3 | CDC25B | CTSB |  |  |  |  |
| FSTL4 | HBEGF | PRDX2 | LDHA |  |  |  |  |
| NOP14 | TIA1 | SCP2 | GRIN2B |  |  |  |  |
| RRS1 | TFF1 | LSS | DNMT3A |  |  |  |  |
| HSF2BP | RECQL4 | PTPRD | TEK |  |  |  |  |
| BET1L | LOXL1 | MMP15 | ALPL |  |  |  |  |
| RHBDD1 | BCOR | MDH1 | HDAC2 |  |  |  |  |
| BCL7C | ZIC2 | ADORA3 | ALK |  |  |  |  |
| ZNF274 | SPINK5 | VAV1 | PRKAR1A |  |  |  |  |
| NT5DC1 | SLC5A7 | F2RL3 | TLR3 |  |  |  |  |
| XIRP2 | BDKRB1 | NT5C2 | TH |  |  |  |  |
| MPP7 | PADI4 | GABRD | FLT1 |  |  |  |  |
| PGBD1 | FABP1 | FKBP1A | JAK3 |  |  |  |  |
| TTLL6 | PAPPA | LDHB | CALR |  |  |  |  |
| SPNS2 | IL18R1 | ADAMTS1 | ATP2A2 |  |  |  |  |
| DYDC1 | THY1 | ZIC1 | SRC |  |  |  |  |
| MICAL3 | PPOX | PDHB | ADAM17 |  |  |  |  |
| KCTD12 | TJP1 | MYO6 | FLT4 |  |  |  |  |
| SEC22B | SIX3 | TMPRSS2 | ZAP70 |  |  |  |  |
| GPKOW | YARS2 | NR4A1 | CXCR4 |  |  |  |  |
| PRICKLE4 | SNAI1 | SLC5A6 | NFKB1 |  |  |  |  |
| ARHGAP42 | UCP3 | POLB | TGFB2 |  |  |  |  |
| ANKRA2 | GMPPB | ATF4 | TGFBR2 |  |  |  |  |
| ZNF804A | EMP2 | GNAT2 | ABCB1 |  |  |  |  |
| GIMAP2 | RAD51C | NTN1 | CASR |  |  |  |  |
| MTHFSD | CDX2 | UNG | JAK1 |  |  |  |  |
| GBA3 | SYNGAP1 | IL1R2 | PIK3CD |  |  |  |  |
| SPINK7 | EFTUD2 | EPHA3 | PRKCD |  |  |  |  |
| HSPB11 | AMBP | CDK7 | MTOR |  |  |  |  |
| EGFL8 | ADAMTSL1 | NNT | MAP2K1 |  |  |  |  |
| MAGEC2 | CYSLTR1 | SPTBN1 | RET |  |  |  |  |
| CPLX3 | PEX5 | FHL2 | ERBB4 |  |  |  |  |
| ZKSCAN4 | PEX2 | COX4I1 | IGF1R |  |  |  |  |
| TCTE1 | COL5A2 | ALDH1A1 | FGFR2 |  |  |  |  |
| ABHD16A | PLCE1 | ADAM12 | FGFR1 |  |  |  |  |
| TTC29 | ALG8 | ARHGEF2 | FGFR3 |  |  |  |  |
| PCDHA10 | SUMF1 | MAP2K4 | PDGFRA |  |  |  |  |
| TEKT5 | LAMP1 | SERPIND1 | PDGFRB |  |  |  |  |
| SH2D3A | LRPPRC | HMGCL | AKT3 |  |  |  |  |
| TXNDC2 | CFP | ERN1 | NTRK2 |  |  |  |  |
| SPAG17 | FABP3 | SSTR3 | AKT2 |  |  |  |  |
| UNC119B | LTBP3 | ALDH1A3 | EZH2 |  |  |  |  |
| TTC30B | TFAM | LAMA4 | DDC |  |  |  |  |
| TRIM40 | CX3CL1 | FPR2 | FZD4 |  |  |  |  |
| CAMKMT | DOCK8 | SUCLG1 | ACVR1 |  |  |  |  |
| PPP1R18 | GDF6 | P2RY2 | DBH |  |  |  |  |
| DMWD | RREB1 | NCOA3 | ITGA2B |  |  |  |  |
| CCDC91 | TCOF1 | PTS | COMT |  |  |  |  |
| ZNF350 | SKIV2L | CCKAR | VDR |  |  |  |  |
| PDZD3 | ARX | SET | AGTR1 |  |  |  |  |
| LUZP1 | SNAI2 | GJA8 | HRAS |  |  |  |  |
| HORMAD2 | OCA2 | EFNB1 | BCR |  |  |  |  |
| CAPN15 | SUFU | PLOD2 | PTEN |  |  |  |  |
| PHRF1 | SOCS3 | DHODH | TLR2 |  |  |  |  |
| TNP1 | ITGA8 | EEF1A2 | TLR4 |  |  |  |  |
| KCNRG | MTRR | ILK | ACE |  |  |  |  |
| RLN1 | SLCO2A1 | ANGPTL3 | CFTR |  |  |  |  |
| DENND1B | PGF | STS | TGFB1 |  |  |  |  |
| ADM2 | MYOCD | PSMA6 | TNF |  |  |  |  |
| ANKS3 | IGFBP1 | DCX | TERT |  |  |  |  |
| PGAP2 | ANKRD1 | PPM1D | RPS6KA3 |  |  |  |  |
| SPATA16 | PPBP | PPIA | ATR |  |  |  |  |
| SSPN | CDKN3 | LIG1 | LCK |  |  |  |  |
| MFHAS1 | RNASEH2A | PITX1 | CDK6 |  |  |  |  |
| IFNL1 | VANGL1 | RIPK2 | MAP2K2 |  |  |  |  |
| MUC3A | NUP107 | XPC | CDH2 |  |  |  |  |
| RCN3 | SMAD7 | NEDD4L | EPHB4 |  |  |  |  |
| EPPIN | TCAP | TRPS1 | DPYD |  |  |  |  |
| EXOC1 | CEACAM5 | PYCR1 | TYK2 |  |  |  |  |
| GSTCD | MBTPS2 | TRPA1 | CDK5 |  |  |  |  |
| CDC123 | SLC26A4 | GFPT1 | RAD51 |  |  |  |  |
| ACP3 | HIRA | SLIT2 | ADAM10 |  |  |  |  |
| STBD1 | FBN2 | TYRP1 | FLT3 |  |  |  |  |
| ADPRH | SDC1 | XPA | SLC2A1 |  |  |  |  |
| DPCD | FOXG1 | IL5RA | BTK |  |  |  |  |
| AHRR | FHIT | CACNB2 | MYLK |  |  |  |  |
| SLC39A2 | CCR8 | GP9 | CREBBP |  |  |  |  |
| TEKT3 | CD2AP | SOX5 | HGF |  |  |  |  |
| CSN1S1 | DNASE1L3 | TPT1 | TGFBR1 |  |  |  |  |
| BRWD3 | MLXIPL | UQCRFS1 | PIK3CA |  |  |  |  |
| PSG2 | MYOC | VIPR1 | CTNNB1 |  |  |  |  |
| CEP89 | AREG | WNT10A | PTPN11 |  |  |  |  |
| RBFOX3 | RBCK1 | CAST | BRAF |  |  |  |  |
| SRY | GDF2 | ACADS | KIT |  |  |  |  |
| IFT20 | IKZF3 | ALDH5A1 | ABL1 |  |  |  |  |
| BCORL1 | CARD14 | NEDD4 | ATM |  |  |  |  |
| NHLRC2 | ABCG8 | NDUFS7 | PSEN1 |  |  |  |  |
| ASXL3 | CLDN2 | XPO1 | JAK2 |  |  |  |  |
| CD24 | AARS2 | NDUFS1 | TP53 |  |  |  |  |
| UQCC2 | PTX3 | VDAC1 | INSR |  |  |  |  |
| ARSL | CD209 | ACADM | DNMT1 |  |  |  |  |
| RNPC3 | DNASE1 | ABCC4 | BCHE |  |  |  |  |
| H2AX | PLEC | CISH | DRD2 |  |  |  |  |
| NSD2 | TOLLIP | HMGA1 | F10 |  |  |  |  |
| CEP104 | KAT6B | FANCL | PRKG1 |  |  |  |  |
| CCL27 | NAT2 | SPRY2 | NOS1 |  |  |  |  |
| NARS1 | ATP12A | SLC4A4 | CASP1 |  |  |  |  |
| IFT52 | RAG2 | BARD1 | PIK3R1 |  |  |  |  |
| HES7 | RAPSN | IGFBP7 | RAC2 |  |  |  |  |
| RARS1 | FOXC1 | YWHAQ | ITGB2 |  |  |  |  |
| TRMT10C | ATP13A2 | LOXL2 | NOTCH2 |  |  |  |  |
| MESP2 | AGTR2 | NRP1 | LEPR |  |  |  |  |
| IL31 | VPS35 | HSD17B4 | NGF |  |  |  |  |
| IBA57 | CD86 | FH | KCNQ1 |  |  |  |  |
| MYO1H | PLOD1 | CRAT | F12 |  |  |  |  |
| CEP85L | IRF4 | AMT | SYK |  |  |  |  |
| CTC1 | HBA1 | PIN1 | EP300 |  |  |  |  |
| TANGO2 | ATG16L1 | TRAF6 | SMO |  |  |  |  |
| MLN | CCL20 | DSC2 | PFKM |  |  |  |  |
| EPRS1 | FARSB | CPB2 | PTCH1 |  |  |  |  |
| KIAA0586 | SLC6A14 | MYH14 | CBL |  |  |  |  |
| KIAA1109 | POT1 | AMPD1 | PTPRC |  |  |  |  |
| DEFB4A | U2AF1 | SEMA4D | SOD2 |  |  |  |  |
| LACC1 | SELL | GCDH | CXCR2 |  |  |  |  |
| DNAJB13 | CSF1 | ETFA | PLA2G7 |  |  |  |  |
| DNAH7 | CARD9 | LAMB3 | VPS33A |  |  |  |  |
| UFD1 | FCGR3B | RHAG | NBAS |  |  |  |  |
| MYRF | SOST | GALNT3 | CXCL5 |  |  |  |  |
| SPEF2 | CAV3 | HELLS | GNRH1 |  |  |  |  |
| DNAAF5 | SURF1 | ROS1 | PF4 |  |  |  |  |
| TMEM216 | IL3 | RAD54L | NEB |  |  |  |  |
| AHDC1 | PRL | ETFDH | G6PC3 |  |  |  |  |
| CCDC103 | LZTR1 | NGFR | A2ML1 |  |  |  |  |
| CCL18 | KRT7 | SEMA3A | NOP10 |  |  |  |  |
| YARS1 | DNAH11 | MYH10 | FLCN |  |  |  |  |
| CCDC65 | TBX20 | SERPINI1 | DNAL1 |  |  |  |  |
| INSL6 | POLR1C | CHRNA7 | GRP |  |  |  |  |
| LRRC56 | CITED2 | ASL | SERPINB1 |  |  |  |  |
| RAP2C | SOD3 | ANTXR1 | LRBA |  |  |  |  |
| VEPH1 | MMACHC | TMPO | SLC26A9 |  |  |  |  |
| GTF3A | NSD1 | NOTCH4 | XK |  |  |  |  |
| ZNF189 | GHRL | KCNE1 | PECAM1 |  |  |  |  |
| NETO1 | TREM2 | ISL1 | IL15 |  |  |  |  |
| FBXW2 | NODAL | ITPR3 | ATP8B1 |  |  |  |  |
| FAM120B | CD34 | LAT | IL7 |  |  |  |  |
| AP1G2 | LCN2 | FADD | NRTN |  |  |  |  |
| KHDRBS2 | RETN | PTGER4 | FKRP |  |  |  |  |
| ZNF277 | IL9 | ITGA2 | CERS1 |  |  |  |  |
| ZDHHC21 | CXCR1 | EIF4EBP1 | POLR2F |  |  |  |  |
| TRIM55 | CEACAM3 | LPAR1 | NPHP4 |  |  |  |  |
| AVEN | OFD1 | FURIN | FLG |  |  |  |  |
| SEC22A | BSCL2 | CLCN2 | GLE1 |  |  |  |  |
| KMT5A | MGP | AGA | SP110 |  |  |  |  |
| TFCP2L1 | THPO | OTX2 | IFT140 |  |  |  |  |
| UCN3 | PLP1 | CD151 | SMN1 |  |  |  |  |
| SVEP1 | SPINK1 | HSD11B2 | DNAH5 |  |  |  |  |
| CDH26 | ASXL1 | DSG2 | MKS1 |  |  |  |  |
| SPCS3 | TBX1 | PTPRO | SCGB1A1 |  |  |  |  |
| LYPLAL1 | CLCN5 | GSS | RNASE3 |  |  |  |  |
| CLDN23 | ACTC1 | CTNND1 | INPP5E |  |  |  |  |
| TTLL1 | HLA-DQA1 | SKI | INF2 |  |  |  |  |
| SYCP1 | HTT | ABCB6 | CEP290 |  |  |  |  |
| HSCB | NPC2 | TNNC1 | EPO |  |  |  |  |
| ZKSCAN5 | UMOD | PTGDS | GPT |  |  |  |  |
| RHOBTB3 | CSF2 | CDON | SFTPA2 |  |  |  |  |
| MED7 | MEFV | ADORA2A | RTEL1 |  |  |  |  |
| RIMBP2 | HNF1B | TP73 | KCNJ9 |  |  |  |  |
| FAM172A | GDAP1 | CD22 | ADAM23 |  |  |  |  |
| TSPAN14 | IL18 | CTSA | AKAP8 |  |  |  |  |
| MED24 | NAGLU | UBA1 | CHRNB3 |  |  |  |  |
| PAMR1 | CCL5 | TWIST1 | FMO4 |  |  |  |  |
| TMSB10 | CCL11 | EXT2 | ARF4 |  |  |  |  |
| ZNF184 | FOXF1 | SCARB1 | GNGT1 |  |  |  |  |
| GNL1 | HLA-DQB1 | FTO | CYP3A43 |  |  |  |  |
| TSPAN4 | IL13 | MAF | PRSS21 |  |  |  |  |
| OSBPL11 | SFTPA1 | MOG | PTGR1 |  |  |  |  |
| FAIM2 | MUC5B | MAG | TRPC5 |  |  |  |  |
| ATP10B | SFTPC | POU5F1 | TGFB1I1 |  |  |  |  |
| FOXD1 | MAP4K1 | MC4R | P2RY6 |  |  |  |  |
| AKAP3 | CDC20 | MSX1 | SLC38A2 |  |  |  |  |
| FGD6 | P2RX1 | WNT1 | C4BPB |  |  |  |  |
| GPX5 | NFATC4 | UCP2 | ASH2L |  |  |  |  |
| GNB1L | MAP3K14 | THBS2 | EDEM1 |  |  |  |  |
| BCL7A | PDIA3 | CA4 | GNA12 |  |  |  |  |
| NUDT21 | CUL5 | DHCR24 | MORF4L1 |  |  |  |  |
| ZGPAT | SMYD2 | CYP21A2 | ERP44 |  |  |  |  |
| PWP2 | LPAR3 | TACR1 | ADAM15 |  |  |  |  |
| GOLGA3 | JAM3 | HABP2 | PDE4DIP |  |  |  |  |
| KRT33B | PAM | CYP7B1 | CACNA2D3 |  |  |  |  |
| CUTA | PPAT | PAX8 | RNF8 |  |  |  |  |
| SCGB2A1 | SLC1A5 | KRT1 | DCTD |  |  |  |  |
| ROPN1L | FGF13 | TJP2 | SSTR4 |  |  |  |  |
| NPDC1 | SIRPA | TCF7L2 | CAP1 |  |  |  |  |
| RBMS3 | CBX5 | SLC2A9 | CNN1 |  |  |  |  |
| ABLIM3 | NCK1 | DAG1 | P2RX6 |  |  |  |  |
| NKRF | NCOR2 | ERCC4 | PTTG1 |  |  |  |  |
| MFSD1 | FSHB | AMACR | ACTR3 |  |  |  |  |
| EFS | FZD8 | PSMB9 | C4BPA |  |  |  |  |
| NRAP | SSTR1 | PKLR | S100A11 |  |  |  |  |
| DNAH2 | SLC7A11 | MST1 | SLC22A8 |  |  |  |  |
| TEKT1 | SLC25A11 | SOX2 | TRIB3 |  |  |  |  |
| TBX10 | DFFA | HDAC8 | RNF13 |  |  |  |  |
| RBM6 | PANX1 | HRH1 | KLK11 |  |  |  |  |
| VGLL4 | LIAS | STAR | RBL1 |  |  |  |  |
| CKMT1B | SPHK2 | APRT | SLC27A6 |  |  |  |  |
| FCHSD2 | TSPAN7 | SLC19A1 | CMKLR1 |  |  |  |  |
| PPP1R3B | CPA6 | PTHLH | RGS2 |  |  |  |  |
| LXN | PLAGL1 | MRAS | PECR |  |  |  |  |
| MAMLD1 | MAD2L2 | KCNA5 | OSBPL2 |  |  |  |  |
| SUPT20H | NAT1 | AQP4 | LEFTY1 |  |  |  |  |
| VTA1 | PSMD7 | BIRC3 | ALOX15B |  |  |  |  |
| NIPA2 | TICAM1 | AGRN | COX4I2 |  |  |  |  |
| KCTD10 | LAMA5 | PPT1 | CCT7 |  |  |  |  |
| SSNA1 | RIPK4 | FANCD2 | RFX2 |  |  |  |  |
| RRP1B | PTPRG | POLD1 | CUL4A |  |  |  |  |
| MLEC | MDK | APAF1 | GPRC5A |  |  |  |  |
| RAB15 | RAN | TPM2 | LIMS2 |  |  |  |  |
| HIRIP3 | CXADR | PMM2 | SIK3 |  |  |  |  |
| ASB1 | PSMD4 | GYPA | RXRG |  |  |  |  |
| NAGPA | TRPV3 | SMC3 | TRPV2 |  |  |  |  |
| PIDD1 | ATF3 | PML | SPINT1 |  |  |  |  |
| KRT23 | SKP2 | PRDM16 | KIRREL2 |  |  |  |  |
| CELA1 | PSMC3 | TGFA | SLC7A8 |  |  |  |  |
| FKBPL | BTC | MASP2 | LRIG1 |  |  |  |  |
| HOPX | GLRX | TPM3 | TP53I3 |  |  |  |  |
| CSTF1 | C3AR1 | PTPN2 | CRTC1 |  |  |  |  |
| MRPS27 | BCAR1 | RIT1 | RASGRF1 |  |  |  |  |
| LECT2 | SIX6 | CHRNA1 | HOXA2 |  |  |  |  |
| FCHO2 | CKM | RRAS2 | KIFAP3 |  |  |  |  |
| ADAMTSL3 | UCP1 | TBX2 | SFRP2 |  |  |  |  |
| PLPP3 | SNRPB | F2RL1 | BAIAP2 |  |  |  |  |
| PLAGL2 | WNT2 | TKT | CLIC1 |  |  |  |  |
| LST1 | VEGFB | IRF3 | KLK5 |  |  |  |  |
| SGMS2 | DDX11 | TGM1 | SMAD5 |  |  |  |  |
| KCTD13 | CUL1 | DLL4 | FABP7 |  |  |  |  |
| DNAH17 | ANK3 | ANGPT1 | CCNG1 |  |  |  |  |
| CHID1 | TIE1 | SLC7A7 | FMO5 |  |  |  |  |
| GSDMA | GATAD2B | ALAD | RALGDS |  |  |  |  |
| TUT1 | ORAI1 | FXN | ITGB8 |  |  |  |  |
| DEFA6 | NFIB | SLC40A1 | ST8SIA2 |  |  |  |  |
| DHDH | JARID2 | MSR1 | KRT6B |  |  |  |  |
| MYOZ1 | POLR1A | LAMA3 | HOMER2 |  |  |  |  |
| PPHLN1 | PAX7 | PRODH | NME7 |  |  |  |  |
| EEFSEC | FUT8 | KDM6A | CYP2A13 |  |  |  |  |
| TCF19 | ATP6V0A4 | STXBP2 | WASL |  |  |  |  |
| GTDC1 | CYP51A1 | LAMB2 | S100A2 |  |  |  |  |
| BAG6 | CEACAM1 | FHL1 | SOCS5 |  |  |  |  |
| GEMIN5 | TAC3 | CRYAA | DDX20 |  |  |  |  |
| H1-4 | TPSAB1 | CRKL | TPX2 |  |  |  |  |
| RNF185 | SLCO1B3 | BAP1 | BNIP3 |  |  |  |  |
| ADAMTSL5 | ACO1 | TBX21 | GPC5 |  |  |  |  |
| KLRC2 | ADAMTS2 | DDX58 | ARHGDIB |  |  |  |  |
| DNAH10 | NID1 | TGIF1 | SERPINA4 |  |  |  |  |
| GULP1 | SPRY4 | GHR | CDK12 |  |  |  |  |
| IL17C | PROK2 | MS4A1 | SSTR5 |  |  |  |  |
| WBP11 | MGAT2 | STAT5A | KDM5A |  |  |  |  |
| BNC1 | DDX1 | COL18A1 | FRS2 |  |  |  |  |
| RNF150 | HGD | CD59 | BNIP3L |  |  |  |  |
| PPP1R21 | RTN4 | AVP | TRAF5 |  |  |  |  |
| LARS1 | AMH | ERCC1 | ATP1B2 |  |  |  |  |
| PTCHD1 | TNNT3 | ABCC9 | TNFRSF10D |  |  |  |  |
| CSNK1A1L | ARNT | XDH | ID3 |  |  |  |  |
| LYRM7 | MANBA | MYCN | CPA3 |  |  |  |  |
| FNIP1 | CHRNB4 | CD81 | ELAVL2 |  |  |  |  |
| P4HTM | LEFTY2 | SLC26A3 | SPRY1 |  |  |  |  |
| SERPINB4 | SPINT2 | SH2D1A | CABIN1 |  |  |  |  |
| GUCA2A | HUWE1 | PPP1CB | RPL19 |  |  |  |  |
| THSD7A | LRP4 | GJA5 | UGT2B17 |  |  |  |  |
| MNS1 | ENPP3 | SMC1A | UBA7 |  |  |  |  |
| PHF11 | A4GALT | CLU | IGF2BP3 |  |  |  |  |
| IFITM5 | PDE3B | ELOVL4 | SMARCA5 |  |  |  |  |
| CDHR3 | AKR1A1 | ITGAL | TRIM27 |  |  |  |  |
| SAA2 | TNFRSF17 | NEK9 | WNT9B |  |  |  |  |
| SYNM | NTF4 | HADHA | PTGER1 |  |  |  |  |
| LINGO2 | APOC2 | LIPC | CREM |  |  |  |  |
| VARS1 | ADNP | SERPINH1 | MTHFD1L |  |  |  |  |
| NUP188 | FGF9 | ABCG5 | KDM2B |  |  |  |  |
| GSDMB | ETFB | HNRNPA2B1 | PSME2 |  |  |  |  |
| SASS6 | COX10 | CLCN7 | UBE2J1 |  |  |  |  |
| ARMC2 | FOXM1 | CD27 | PADI3 |  |  |  |  |
| TLX2 | CGA | CR1 | STC1 |  |  |  |  |
| CENPB | DCK | EFEMP2 | SLC24A5 |  |  |  |  |
| TAS2R38 | SORT1 | AFP | MT2A |  |  |  |  |
| ANKRD2 | CHRNE | PPARGC1A | CDIPT |  |  |  |  |
| FUT4 | NLRP12 | CD38 | SCARF2 |  |  |  |  |
| EVPL | RPL15 | ATRX | GART |  |  |  |  |
| ZNF292 | KIF22 | GALNS | HSPH1 |  |  |  |  |
| C12orf57 | LPP | TRPV1 | PTN |  |  |  |  |
| WDPCP | XRCC5 | WFS1 | SH3GL3 |  |  |  |  |
| CNTLN | ARID1A | ANXA5 | TNFSF14 |  |  |  |  |
| KIAA1549 | NEUROD1 | CCR1 | EFHC1 |  |  |  |  |
| LUC7L2 | PSMA4 | DCTN1 | RNF5 |  |  |  |  |
| ABO | GBF1 | GP1BA | AP2B1 |  |  |  |  |
| COQ8A | MYBPC1 | SCN4A | HAND1 |  |  |  |  |
| AMER1 | BSG | PTH | CYP2F1 |  |  |  |  |
| IL9R | MAFB | SETD2 | KLF15 |  |  |  |  |
| POMGNT2 | TYROBP | PRPS1 | PROZ |  |  |  |  |
| DDRGK1 | ABCG1 | MYOD1 | ICMT |  |  |  |  |
| RACK1 | ACAD9 | ITCH | PARD3 |  |  |  |  |
| INTU | M6PR | CTSG | MAP1LC3A |  |  |  |  |
| TGDS | NPC1L1 | LOX | POLI |  |  |  |  |
| TANC2 | FABP4 | CR2 | PLCZ1 |  |  |  |  |
| MEPE | MSH3 | ETV6 | DLG2 |  |  |  |  |
| IFT27 | CEBPB | CD14 | IGSF1 |  |  |  |  |
| KLHL40 | PGM3 | F13A1 | RIPK3 |  |  |  |  |
| OMP | UBE2A | RAG1 | FIBP |  |  |  |  |
| CCL24 | PEX14 | ERCC6 | RAB11B |  |  |  |  |
| CLUAP1 | REST | GZMB | AGRP |  |  |  |  |
| TMEM138 | GAL | CCR3 | GABARAPL1 |  |  |  |  |
| BLOC1S1 | MCOLN1 | KL | LAP3 |  |  |  |  |
| CCL13 | IL12RB2 | NPHS1 | GSTZ1 |  |  |  |  |
| MPEG1 | DYSF | CHD7 | SCAP |  |  |  |  |
| CD52 | PICALM | MYBPC3 | PSMC5 |  |  |  |  |
| WDR73 | ACD | IFIH1 | FMOD |  |  |  |  |
| AHSP | S100A4 | TLR9 | ASRGL1 |  |  |  |  |
| APLN | NOD1 | CD8A | LUM |  |  |  |  |
| RBM20 | VTN | PROM1 | DGKQ |  |  |  |  |
| BPIFA1 | FOXC2 | CPS1 | AKAP12 |  |  |  |  |
| PHB1 | GAB2 | CD28 | FGL2 |  |  |  |  |
| SAMD9 | NFIX | IL1R1 | DKK3 |  |  |  |  |
| B9D1 | MYH3 | HNRNPA1 | MBD4 |  |  |  |  |
| CEP83 | BCL2L11 | BMP2 | MARCO |  |  |  |  |
| DNAJC30 | NDUFV2 | TARDBP | LIPF |  |  |  |  |
| LACTB | MADD | EPHX1 | SLC35A3 |  |  |  |  |
| TMEM237 | EGR1 | HP | CRABP1 |  |  |  |  |
| USB1 | PODXL | SELP | FCAR |  |  |  |  |
| PKD1L1 | DSG1 | STAT4 | TUSC3 |  |  |  |  |
| IRGM | POMT2 | FGF23 | ARFGEF2 |  |  |  |  |
| IFT80 | BAK1 | MEN1 | LTBR |  |  |  |  |
| RSPH3 | ARID1B | FGF2 | TMPRSS15 |  |  |  |  |
| RNASEH2B | FCER2 | VHL | CLCA2 |  |  |  |  |
| NKX2-6 | PDGFA | TYMP | EREG |  |  |  |  |
| DNAH1 | APEX1 | MYH6 | LGMN |  |  |  |  |
| DNAAF3 | BGN | GANAB | ID1 |  |  |  |  |
| DNAAF1 | TLR6 | TNFRSF11A | TXNL4A |  |  |  |  |
| DNAAF2 | EPX | PINK1 | RING1 |  |  |  |  |
| CCDC39 | MTM1 | NPPA | PIGR |  |  |  |  |
| CCDC40 | COL9A3 | MMP12 | NPAS2 |  |  |  |  |
| DZIP1L | DNAJC5 | FOXP3 | CDK13 |  |  |  |  |
| GDF1 | EWSR1 | NKX2-1 | SLAMF7 |  |  |  |  |
| GARS1 | ASCL1 | SMAD9 | SIM1 |  |  |  |  |
| OSGEPL1 | NDUFS2 | HEXA | RAD52 |  |  |  |  |
| NLRP10 | SLC29A3 | MYH11 | GPSM2 |  |  |  |  |
| RGS8 | GNE | TBX5 | DPP10 |  |  |  |  |
| AFAP1L2 | WIPF1 | ACTG2 | PLIN2 |  |  |  |  |
| GMPR2 | GH1 | CCR5 | FLOT1 |  |  |  |  |
| DHX15 | HAVCR2 | SOX10 | ICAM3 |  |  |  |  |
| TAF7 | GREM1 | NEFL | AP2S1 |  |  |  |  |
| THAP5 | NDUFS4 | ABCA4 | SLC26A8 |  |  |  |  |
| ERN2 | FMR1 | PRTN3 | FGF18 |  |  |  |  |
| GPSM1 | TPP1 | HBB | TUBA1B |  |  |  |  |
| GZMK | BAG3 | FCGR2A | MFAP2 |  |  |  |  |
| ASMT | XRCC1 | F5 | SLC6A20 |  |  |  |  |
| ARL4A | SEMA3C | PARN | UBE4B |  |  |  |  |
| MOXD1 | SOCS1 | PKD1 | RGS6 |  |  |  |  |
| TNIP2 | BCL6 | PTK6 | NTM |  |  |  |  |
| TSPAN5 | IFNGR2 | ADCY3 | MCAM |  |  |  |  |
| STK32C | SLC18A3 | ITGB5 | TBR1 |  |  |  |  |
| CDH20 | BCL10 | TTK | SLC3A2 |  |  |  |  |
| ARHGEF17 | COX5A | PRMT1 | HHEX |  |  |  |  |
| SREK1 | NEK1 | YWHAZ | RB1CC1 |  |  |  |  |
| HOXD11 | SPG7 | CA1 | F13B |  |  |  |  |
| MAFK | SLC22A4 | ADH5 | PGAP1 |  |  |  |  |
| DHX29 | TIMP2 | PGD | LY96 |  |  |  |  |
| PARP6 | CRH | HSP90AB1 | IL13RA1 |  |  |  |  |
| AIFM3 | NHP2 | ATP6V1B2 | MPDU1 |  |  |  |  |
| NAPA | LGALS3 | SIRT2 | NAGS |  |  |  |  |
| TAS2R1 | CHIT1 | RRM1 | HNRNPU |  |  |  |  |
| KDM4D | OPA1 | EPB41 | CYP46A1 |  |  |  |  |
| FEZ2 | COL11A1 | AK1 | NDUFB3 |  |  |  |  |
| AQP8 | CD274 | HDAC5 | FANCF |  |  |  |  |
| TRO | MB | CHN1 | COQ9 |  |  |  |  |
| CXXC4 | OPTN | TCF12 | PTF1A |  |  |  |  |
| SETD3 | PHOX2B | TUBG1 | TNFRSF18 |  |  |  |  |
| DUSP14 | NLRP1 | RFC1 | JUNB |  |  |  |  |
| CRISPLD2 | CLN3 | SPHK1 | APCS |  |  |  |  |
| AMOTL1 | IL12A | GRM7 | GDF11 |  |  |  |  |
| TACC2 | SEC63 | ADCY6 | PUF60 |  |  |  |  |
| DDX39A | GCLC | LHCGR | ASAH2 |  |  |  |  |
| ARSK | HAMP | AKR1C4 | NRG3 |  |  |  |  |
| IFNA5 | HSPB8 | DUSP1 | TRNT1 |  |  |  |  |
| OSBPL6 | SDHD | KLK1 | PYCARD |  |  |  |  |
| SLC5A12 | TG | FST | TRIP13 |  |  |  |  |
| PHF21B | CST3 | RRM2 | COQ6 |  |  |  |  |
| MRPL40 | NPHP1 | GABRA2 | IL24 |  |  |  |  |
| PPFIA2 | INVS | MAT1A | TSG101 |  |  |  |  |
| PES1 | CALCA | ROCK2 | SP7 |  |  |  |  |
| TEKT2 | BMP6 | DIABLO | MAP1LC3B |  |  |  |  |
| MED30 | LITAF | CYP2B6 | ADAMTS17 |  |  |  |  |
| CYP2A7 | IL12B | MDH2 | CHST14 |  |  |  |  |
| TXNRD3 | SLC34A1 | LAMA1 | AIM2 |  |  |  |  |
| SSR3 | IDUA | CSK | CCBE1 |  |  |  |  |
| HSPA12A | SLC17A5 | FMO3 | SPRED1 |  |  |  |  |
| ZWINT | NPPB | MAP3K5 | SETDB1 |  |  |  |  |
| KLHL22 | SFTPD | SIK1 | JAM2 |  |  |  |  |
| TAX1BP3 | HFE | PRKAA1 | TWIST2 |  |  |  |  |
| OTUD4 | S1PR5 | YWHAB | TRH |  |  |  |  |
| IP6K3 | PARP2 | CLDN1 | ELAVL1 |  |  |  |  |
| RNF220 | USP10 | MMP10 | CHKA |  |  |  |  |
| TNNC2 | HPN | GNRHR | ALG2 |  |  |  |  |
| BTN3A2 | EZH1 | TALDO1 | KISS1 |  |  |  |  |
| EYA2 | USP14 | PTGDR | MSLN |  |  |  |  |
| RAPGEF6 | DGKA | KAT2B | SLC35A2 |  |  |  |  |
| OSCAR | SQLE | HMOX2 | MLYCD |  |  |  |  |
| ANAPC5 | PPP4C | HYAL1 | TERF1 |  |  |  |  |
| EDC4 | PPM1A | SIRT3 | SALL2 |  |  |  |  |
| TSSK1B | RPS6KA5 | ITGAV | EARS2 |  |  |  |  |
| NME5 | DYRK2 | WNT3A | IGBP1 |  |  |  |  |
| SIGLEC9 | STAMBP | GLP1R | DGCR8 |  |  |  |  |
| EIF4ENIF1 | HMGCS2 | GLDC | MSH5 |  |  |  |  |
| C1QTNF6 | PPP1CC | ADORA1 | SOX11 |  |  |  |  |
| BTN2A2 | METAP2 | DRD5 | ARNTL |  |  |  |  |
| SLITRK3 | SLC27A2 | PTGIR | KRIT1 |  |  |  |  |
| COL20A1 | MAD2L1 | CTSH | SRF |  |  |  |  |
| RAB11FIP4 | LAMC1 | GABBR1 | ARL6 |  |  |  |  |
| ZKSCAN1 | DOT1L | GHSR | RERE |  |  |  |  |
| CDX1 | P2RX2 | AQP3 | ADAMTSL2 |  |  |  |  |
| MRPL28 | PDE4C | PTPRJ | MEGF10 |  |  |  |  |
| PRUNE2 | KCNH5 | OAT | SPRED2 |  |  |  |  |
| CHD6 | PPP3CB | MSN | AP1S2 |  |  |  |  |
| HEPHL1 | VIPR2 | ECHS1 | PMS1 |  |  |  |  |
| ANGPTL2 | WNT5B | USP9X | STAG3 |  |  |  |  |
| ARFRP1 | ATP2B4 | MAN1B1 | LEMD3 |  |  |  |  |
| NGB | PFKP | MYLK2 | CNBP |  |  |  |  |
| TIGAR | TUBA8 | RAB11A | PIGV |  |  |  |  |
| ERRFI1 | PIAS1 | GNAO1 | RIN2 |  |  |  |  |
| FOXL1 | SLC15A1 | TRAF3 | PACS1 |  |  |  |  |
| TCEAL1 | HIPK2 | FZD5 | AFF2 |  |  |  |  |
| DCD | ADCY9 | ADSL | SLC30A8 |  |  |  |  |
| ZNF74 | GRK6 | GPI | LRIG2 |  |  |  |  |
| TAF1C | GGPS1 | PI4KA | ANXA6 |  |  |  |  |
| SCG5 | HSD17B2 | GLS | FABP2 |  |  |  |  |
| DBP | SHMT1 | LFNG | HPSE2 |  |  |  |  |
| RCE1 | PFKL | MYH2 | AGGF1 |  |  |  |  |
| COPS7A | KIF5B | IRAK3 | LMO2 |  |  |  |  |
| PPP1R14C | SDC2 | FOLH1 | MIP |  |  |  |  |
| MYBPH | NR2C2 | KCNJ6 | TNFRSF4 |  |  |  |  |
| NCKIPSD | NR4A3 | SCO1 | VPS33B |  |  |  |  |
| RASGRP4 | TLE1 | ABCC3 | CD1D |  |  |  |  |
| GPR15 | KPNA2 | CDH3 | MRPS22 |  |  |  |  |
| CLEC4A | MBTPS1 | CTNNA1 | AIMP2 |  |  |  |  |
| RMI1 | HCRTR1 | SLC11A2 | RPL26 |  |  |  |  |
| SPATA13 | SMURF1 | MSX2 | KLRD1 |  |  |  |  |
| PLRG1 | CTSZ | SLC25A1 | FSCN1 |  |  |  |  |
| MYCL | FAP | IRF7 | PKP1 |  |  |  |  |
| SPAG6 | INHBA | CUL3 | BANF1 |  |  |  |  |
| TRIM26 | TXN2 | CAMK2G | SSB |  |  |  |  |
| ECD | PKN2 | CTSC | TRPC1 |  |  |  |  |
| PRDM9 | PAK6 | TPH2 | TFEB |  |  |  |  |
| SOX1 | CYP26A1 | GPX4 | NADSYN1 |  |  |  |  |
| DEFA3 | HDAC7 | PLCG1 | PSMC4 |  |  |  |  |
| PDLIM3 | MCM5 | CEBPA | RHCE |  |  |  |  |
| ZBTB38 | PVR | PLCB4 | CREB3L1 |  |  |  |  |
| CLPTM1L | MGLL | BRD4 | PTGDR2 |  |  |  |  |
| UPK2 | DLX5 | HCFC1 | CD63 |  |  |  |  |
| IFIT3 | CES2 | IHH | PDSS1 |  |  |  |  |
| CIRBP | CHD1 | DDR1 | NXN |  |  |  |  |
| ATP6V1G2 | MMP16 | CYSLTR2 | CCL21 |  |  |  |  |
| AP3M1 | HYOU1 | HPGD | KMT2C |  |  |  |  |
| CDK5RAP1 | ULK1 | AXIN1 | STIL |  |  |  |  |
| RERG | TCF7 | GNA11 | SYN3 |  |  |  |  |
| HNRNPUL1 | SLC44A1 | CTSL | ERLIN2 |  |  |  |  |
| CRLF3 | SLC22A6 | TYMS | SPTB |  |  |  |  |
| APIP | E2F4 | ADORA2B | IAPP |  |  |  |  |
| CXCL14 | ADCY8 | NR1H2 | CAPN10 |  |  |  |  |
| DHX34 | CFD | TGFBR3 | DSCAM |  |  |  |  |
| DNALI1 | CDH15 | HADHB | LIPT1 |  |  |  |  |
| CCL25 | PBRM1 | ANXA2 | DSG3 |  |  |  |  |
| ZFPM1 | FOXA2 | EPHX2 | ADAMTS3 |  |  |  |  |
| CCDC8 | ABCA5 | P4HB | ANK2 |  |  |  |  |
| EBAG9 | PRDX5 | P4HA2 | TDGF1 |  |  |  |  |
| SOX30 | HIF1AN | KAT5 | KCNE2 |  |  |  |  |
| PIGW | CHD4 | NOG | DNTT |  |  |  |  |
| DERL2 | PRSS8 | FYN | RPS26 |  |  |  |  |
| FRMD4A | NUP62 | WNK1 | LTC4S |  |  |  |  |
| IL36G | WNT11 | IDE | ANLN |  |  |  |  |
| TRAPPC10 | PSMB1 | CACNA1D | FBL |  |  |  |  |
| IER3 | GAB1 | CDH5 | LIF |  |  |  |  |
| GPR182 | FZD3 | DDX3X | CD1A |  |  |  |  |
| ESCO1 | S1PR2 | ERCC3 | RP2 |  |  |  |  |
| DAND5 | CBR1 | ZEB1 | SNAP29 |  |  |  |  |
| SETD1B | EIF5A | FUCA1 | PEX3 |  |  |  |  |
| KNSTRN | FZD7 | WNT3 | UBC |  |  |  |  |
| DERL1 | CYP4F2 | WWOX | ANO1 |  |  |  |  |
| PSMA8 | B4GALT1 | C2 | MNX1 |  |  |  |  |
| PIGM | FGF5 | SLC3A1 | DARS2 |  |  |  |  |
| AP2A2 | TRAF2 | GLI1 | MCM3AP |  |  |  |  |
| DRAM2 | KRT6A | HTR2C | ADCYAP1 |  |  |  |  |
| HSD17B13 | HRG | CCND3 | FOXH1 |  |  |  |  |
| P3H2 | EMX2 | TXNRD2 | HOXD13 |  |  |  |  |
| RHOD | SLC30A1 | GDF5 | CCL7 |  |  |  |  |
| CFDP1 | NFIC | GRB2 | TOP3A |  |  |  |  |
| AEBP2 | ATG4B | UBE3A | FKBP10 |  |  |  |  |
| AFF3 | MED15 | DVL1 | PLXND1 |  |  |  |  |
| PRPS1L1 | MPZL1 | CDC25A | CHRND |  |  |  |  |
| SRA1 | NACC1 | LAMC2 | IGFBP5 |  |  |  |  |
| PENK | SCTR | NR1H3 | EIF4H |  |  |  |  |
| NCAPH2 | REV1 | CYP24A1 | PEX19 |  |  |  |  |
| AFAP1 | CHGB | FAH | APOA4 |  |  |  |  |
| CCP110 | KANK1 | SERPING1 | XRCC3 |  |  |  |  |
| MAML2 | CDK20 | ARHGDIA | BPI |  |  |  |  |
| WDR11 | HLA-DOB | RASGRP1 | CDCA7L |  |  |  |  |
| ANOS1 | MCC | KRT14 | RBP3 |  |  |  |  |
| MYNN | ONECUT1 | F2R | IARS2 |  |  |  |  |
| GORAB | TNPO1 | ACVR1B | IGSF3 |  |  |  |  |
| KRT74 | ELAVL4 | ANTXR2 | TRIM63 |  |  |  |  |
| EML4 | PDZK1 | WNT4 | RHD |  |  |  |  |
| TONSL | DYNC1I2 | LYZ | TRIM21 |  |  |  |  |
| SPAG16 | FUCA2 | RPL11 | XRCC2 |  |  |  |  |
| LCOR | HDGF | DHCR7 | SLC30A10 |  |  |  |  |
| MSL3 | DAB2IP | TPI1 | DUOX1 |  |  |  |  |
| TET1 | FAT1 | PRKAR1B | PEX10 |  |  |  |  |
| FHOD3 | CD93 | ENO1 | AGK |  |  |  |  |
| SLX4 | TIMP4 | PTK2 | MX1 |  |  |  |  |
| DEFA5 | GALNT11 | FGF1 | IL1RL1 |  |  |  |  |
| WDR37 | SEMA6A | PAX5 | NTF3 |  |  |  |  |
| SESN2 | NFKBIE | MEF2A | SLC34A3 |  |  |  |  |
| EXOSC10 | RSAD2 | SLC18A2 | FANCE |  |  |  |  |
| TSHZ3 | PSMD11 | DCN | OSM |  |  |  |  |
| MEGF8 | TPM4 | DNM1L | TMEM43 |  |  |  |  |
| VIPAS39 | GTF2H4 | ATF6 | NOX1 |  |  |  |  |
| NBR1 | DEFA1 | CNR1 | BTBD1 |  |  |  |  |
| POC1B | KDELR2 | ENTPD1 | C1D |  |  |  |  |
| DDX39B | GOPC | ETS1 | ASAP2 |  |  |  |  |
| IFT74 | PSMB2 | C1S | COX17 |  |  |  |  |
| FAM126A | SNX9 | KCNJ5 | SNRPF |  |  |  |  |
| SNRNP70 | ADARB2 | OTC | NUMBL |  |  |  |  |
| SHROOM3 | LTB | EPOR | CADM2 |  |  |  |  |
| GLCCI1 | RPLP2 | HTR1A | BMP8A |  |  |  |  |
| SULT1A3 | FMN2 | PTGIS | COL15A1 |  |  |  |  |
| ZBTB24 | PPA2 | TPMT | ABHD2 |  |  |  |  |
| AHNAK | DPH1 | NAMPT | CHD9 |  |  |  |  |
| ALG12 | SIGLEC8 | HSPA8 | EEF1G |  |  |  |  |
| SLC52A2 | FGF16 | SLC25A13 | PPP1R12B |  |  |  |  |
| ATP13A3 | PRDM2 | VEGFC | EXTL2 |  |  |  |  |
| SPG21 | NINJ1 | CTCF | PDCD5 |  |  |  |  |
| THSD4 | CA3 | PAX3 | MRRF |  |  |  |  |
| HLA-DQA2 | ASH1L | PGK1 | SMTN |  |  |  |  |
| DCHS1 | AP3B2 | KEAP1 | HSPA14 |  |  |  |  |
| EVC | COL27A1 | CYP3A5 | DHRS9 |  |  |  |  |
| IBSP | THG1L | KLK3 | NEIL1 |  |  |  |  |
| REG3A | SRPX2 | FANCC | TMOD3 |  |  |  |  |
| CCL1 | ASGR2 | LIFR | NID2 |  |  |  |  |
| GHRH | HERC1 | SCN1A | PIM3 |  |  |  |  |
| CRLF2 | IL17B | AVPR2 | SAFB |  |  |  |  |
| ASTN2 | LGALS2 | VKORC1 | CDC42EP3 |  |  |  |  |
| SPECC1L | ALG6 | TFAP2A | SCAMP2 |  |  |  |  |
| ZNF699 | INSL3 | PDX1 | KCNK5 |  |  |  |  |
| HNRNPH2 | TFF2 | AQP5 | TDRKH |  |  |  |  |
| SIAE | MVP | VCL | SEZ6L |  |  |  |  |
| DCAF8 | VAMP8 | IL2RG | GKN1 |  |  |  |  |
| ARL13B | PSMB3 | CYP11B2 | ABCA13 |  |  |  |  |
| EOGT | CRYGD | RBPJ | ARHGAP32 |  |  |  |  |
| TCTN1 | SPEN | EIF2AK2 | SAT2 |  |  |  |  |
| TTC14 | CLDN18 | FLNB | TSC22D3 |  |  |  |  |
| MMEL1 | SUZ12 | ACTN2 | PLXDC2 |  |  |  |  |
| UPK3A | TBX19 | C5 | MYO1D |  |  |  |  |
| FCRL3 | KCTD1 | GCH1 | PHC3 |  |  |  |  |
| ADGRV1 | SLC35D1 | IGF2R | NIT2 |  |  |  |  |
| NUP205 | ESM1 | SLC12A3 | GNPDA2 |  |  |  |  |
| IL27 | SPPL2A | CYP27B1 | CD53 |  |  |  |  |
| BOLA3 | SNRPA | RHOA | DUSP13 |  |  |  |  |
| CFC1 | PSMC2 | CD44 | PDZD2 |  |  |  |  |
| CCL26 | OBSCN | LMNB1 | SUN1 |  |  |  |  |
| NFKBIL1 | TTC19 | YAP1 | CTNNAL1 |  |  |  |  |
| BCL7B | MEST | PLA2G2A | RGS17 |  |  |  |  |
| GRK2 | FMO2 | HDAC9 | ACAD10 |  |  |  |  |
| IFT43 | PPL | GPX1 | STIM2 |  |  |  |  |
| IL23A | ETS2 | AQP1 | ZDHHC8 |  |  |  |  |
| MAGEL2 | PDCD1LG2 | NRG1 | LAMP3 |  |  |  |  |
| NEK10 | POLR2L | CSTB | NUBP2 |  |  |  |  |
| TMEM231 | ECEL1 | GNB3 | CEBPD |  |  |  |  |
| MUC7 | CAMK1D | MTR | ATF6B |  |  |  |  |
| IL37 | TMSB4X | CDKN1C | UPK1A |  |  |  |  |
| FOXE3 | TET3 | HPRT1 | SYMPK |  |  |  |  |
| CLN6 | FOXL2 | ABCC8 | RECQL5 |  |  |  |  |
| CCL22 | PLEKHM1 | AQP2 | FOXA3 |  |  |  |  |
| DNAH9 | NAB2 | DRD4 | SLC36A1 |  |  |  |  |
| KARS1 | ALG14 | IKZF1 | BTN2A1 |  |  |  |  |
| RSPH9 | HPX | L1CAM | SFRP5 |  |  |  |  |
| CCL17 | PHF1 | IRS1 | USP48 |  |  |  |  |
| DNAH8 | MBD2 | TCF4 | SOBP |  |  |  |  |
| RSPH4A | BCAP31 | SLC22A5 | NLRC5 |  |  |  |  |
| SP140 | APOM | LIG4 | BRWD1 |  |  |  |  |
| FOXJ1 | UPF3B | MITF | GPM6B |  |  |  |  |
| WDR19 | GCM2 | FTL | TSR1 |  |  |  |  |
| TTC21B | WWTR1 | CD55 | STOM |  |  |  |  |
| HYDIN | SYVN1 | ZIC3 | NUCB2 |  |  |  |  |
| DYNC2H1 | CHD8 | PIK3C2A | PPP1R10 |  |  |  |  |
| PKHD1 | MYO1A | ANGPT2 | RBM28 |  |  |  |  |
| GPRC5B | RECK | MECOM | SPDEF |  |  |  |  |
| RGS11 | PDSS2 | ABCC6 | NEBL |  |  |  |  |
| CHRNA10 | PSMC6 | CDKN1B | MAML3 |  |  |  |  |
| AKAP5 | NLGN4X | COL5A1 | ZFP36 |  |  |  |  |
| NUDT12 | SH3PXD2B | TGM2 | LZTS1 |  |  |  |  |
| FLRT2 | CBY1 | ACTN4 | KHDRBS3 |  |  |  |  |
| PMEPA1 | ATP2C2 | NCAM1 | DKK4 |  |  |  |  |
| HERC5 | SAA4 | MUSK | DPY19L2 |  |  |  |  |
| CPXM2 | KLF2 | LBR | RCVRN |  |  |  |  |
| ILF2 | HLA-DOA | GLI2 | SRSF6 |  |  |  |  |
| PRMT6 | RGS5 | HSPG2 | SRRT |  |  |  |  |
| TMPRSS11D | FBXO32 | TLR7 | SLC9A7 |  |  |  |  |
| MYL12A | S100A7 | SCNN1G | FMN1 |  |  |  |  |
| DDT | BHMT | TNFAIP3 | SH3PXD2A |  |  |  |  |
| MGAT5B | HSPE1 | TAP1 | ACR |  |  |  |  |
| TAAR1 | SMPD2 | F11 | GOSR1 |  |  |  |  |
| BTG3 | GNPTG | TNFRSF13B | WLS |  |  |  |  |
| ME3 | APOBEC3G | PITX2 | HOXB5 |  |  |  |  |
| RBBP5 | GTPBP3 | KRT5 | GRK3 |  |  |  |  |
| TRMT2A | MED13 | TAB2 | PDS5A |  |  |  |  |
| ZW10 | PPP1R13L | TSHR | LY86 |  |  |  |  |
| MOCS3 | PITX3 | KRT8 | IK |  |  |  |  |
| ZC3HAV1 | ADAMTS7 | CDKN2B | B3GNT2 |  |  |  |  |
| RPL32 | EPB42 | FGB | PPP1R14A |  |  |  |  |
| HIGD1A | EDN2 | DICER1 | CELF1 |  |  |  |  |
| PLXNA4 | SDHAF2 | TLR1 | ZFP57 |  |  |  |  |
| ANAPC10 | PORCN | IKBKG | SLCO1A2 |  |  |  |  |
| TESK2 | SETD1A | CFI | ABCF2 |  |  |  |  |
| PFDN4 | KMT2E | DKC1 | TRIM31 |  |  |  |  |
| NUDT5 | SIGLEC1 | ZEB2 | ETV5 |  |  |  |  |
| POLR2H | FKBP14 | FGF8 | HAS1 |  |  |  |  |
| SLC45A3 | DSE | ASAH1 | SOX8 |  |  |  |  |
| CCNL1 | PTMA | ERCC2 | RAB4B |  |  |  |  |
| CHI3L2 | CEP55 | NBN | SLC9A3R2 |  |  |  |  |
| ATP6V0D2 | TBX6 | PYGL | DHX37 |  |  |  |  |
| DRG2 | ALG3 | NR4A2 | TNRC6A |  |  |  |  |
| DOC2A | MYH8 | FBLN5 | KIF17 |  |  |  |  |
| NDST2 | MDC1 | SDHA | YBX1 |  |  |  |  |
| SESN1 | SLC4A11 | ACP5 | MYOZ2 |  |  |  |  |
| SERPINB9 | CCNT1 | TGFB3 | MMP23B |  |  |  |  |
| MAS1 | IFRD1 | HSPD1 | HNRNPH1 |  |  |  |  |
| ABCE1 | GCLM | GSTM3 | IL1F10 |  |  |  |  |
| FKBP6 | NPSR1 | ENO2 | SPON1 |  |  |  |  |
| CSPP1 | IL15RA | IL7R | CEACAM7 |  |  |  |  |
| IL1RAPL2 | SLC35A1 | SOX9 | LRRK1 |  |  |  |  |
| PIEZO1 | SYNE2 | SCNN1A | PIGP |  |  |  |  |
| FGF20 | COL13A1 | GATA1 | BST2 |  |  |  |  |
| SPAST | TMC8 | SMAD6 | DUSP19 |  |  |  |  |
| MICA | DGCR2 | CYP27A1 | TRIM39 |  |  |  |  |
| ANKS6 | PLAG1 | DMD | SYN2 |  |  |  |  |
| ELP4 | RBM10 | SH2B3 | PSMD1 |  |  |  |  |
| TTC7A | FGD1 | CUBN | BICD1 |  |  |  |  |
| FKTN | SLC35C1 | ARSA | WDR4 |  |  |  |  |
| WDR35 | SMCHD1 | RAB7A | ADAMTS9 |  |  |  |  |
| PI3 | DIO2 | KNG1 | PSPN |  |  |  |  |
| GAS8 | RFX6 | STX1A | TELO2 |  |  |  |  |
| PACRG | DHX16 | TNFRSF1B | NUDT6 |  |  |  |  |
| SPAG1 | PLEK | ABCB11 | CXCL6 |  |  |  |  |
| ZMYND10 | PIGT | PAX2 | GLRX5 |  |  |  |  |
| HGSNAT | SNCG | LCAT | HLA-DQB2 |  |  |  |  |
| RNASEH2C | MS4A2 | CSF3R | THOC2 |  |  |  |  |
| CLCA4 | CLDN3 | P2RX7 | RIMS1 |  |  |  |  |
| TRAF3IP1 | LCN1 | SLC9A3 | ACKR2 |  |  |  |  |
| SLC26A1 | MCTP2 | PDCD1 | GIP |  |  |  |  |
| SIGLEC5 | CTNNA3 | DES | NPNT |  |  |  |  |
| BNC2 | TRIP4 | GJB2 | CTF1 |  |  |  |  |
| BBS1 | PEX11B | TNFSF11 | KANK2 |  |  |  |  |
| CXCL9 | SLAMF1 | CD79A | RDH10 |  |  |  |  |
| HCRT | TOR1AIP1 | TLR5 | ARMC9 |  |  |  |  |
| CCL4 | TERF2 | SDHB | CCDC22 |  |  |  |  |
| RPGRIP1L | TTC8 | IRF5 | CLEC11A |  |  |  |  |
| FIP1L1 | IFT81 | MPL | NSMF |  |  |  |  |
| ADAM33 | TLR10 | ATP7A | PKDCC |  |  |  |  |
| DCTN4 | VSX2 | ITGAM | CELA3B |  |  |  |  |
| TSLP | SNX10 | F8 | TMEM127 |  |  |  |  |
| IL33 | CCL19 | SOS1 | SHOX |  |  |  |  |
| NPHP3 | FCGR1A | TTN | GPR101 |  |  |  |  |
| CC2D2A | CXCR6 | GFAP | PMEL |  |  |  |  |
| TMEM67 | PCDH15 | FASLG | CEP152 |  |  |  |  |
| MUC5AC | HLA-DRB5 | HLA-A | MIA2 |  |  |  |  |
| CCN2 | DLL3 | ABCB4 | CD1B |  |  |  |  |
| CCL3 | NEUROG3 | CHAT | PMAIP1 |  |  |  |  |
| SH3TC2 | SH3BP2 | FGF10 | BMP10 |  |  |  |  |
| PRKN | KIF3A | MBL2 | LEMD2 |  |  |  |  |
| FAM13A | ITGAE | CFH | SUN2 |  |  |  |  |
| PCDH9 | TBCD | PSAP | TRIM8 |  |  |  |  |
| LRRTM1 | ADGRG6 | ACTA2 | CASZ1 |  |  |  |  |
| ELF3 | PEX13 | TSC1 | PPT2 |  |  |  |  |
| FN3K | ADGRG1 | GATA6 | CDAN1 |  |  |  |  |
| CNOT4 | SRCAP | LEP | PCGF2 |  |  |  |  |
| SERINC1 | GBA2 | BDNF | LGALS9 |  |  |  |  |
| COX6C | MMP21 | MYH7 | DSPP |  |  |  |  |
| EIF2A | CXCR5 | GJB1 | HESX1 |  |  |  |  |
| TRDMT1 | CNTF | GDNF | HIVEP2 |  |  |  |  |
| SLC26A7 | NUP93 | SLC11A1 | COG6 |  |  |  |  |
| PAG1 | TFF3 | ATP7B | SYNPO |  |  |  |  |
| RNF41 | COLQ | SQSTM1 | RBFOX1 |  |  |  |  |
| CDH19 | SIL1 | INS | NIPAL4 |  |  |  |  |
| LILRB3 | FOXRED1 | MFN2 | NCR3 |  |  |  |  |
| USP3 | ZNF423 | RYR1 | FBXL4 |  |  |  |  |
| RNF34 | TBCK | POLG | ABHD11 |  |  |  |  |
| SPAM1 | SERPINA7 | PTPN22 | MAVS |  |  |  |  |
| ANAPC2 | ORMDL3 | FBN1 | CCHCR1 |  |  |  |  |
| BAZ1A | SOX18 | NLRP3 | SLMAP |  |  |  |  |
| ABCB9 | KLHL7 | NPC1 | NIPA1 |  |  |  |  |
| S100P | MSMB | NCF4 | CD1E |  |  |  |  |
| MAPK15 | CRELD1 | HLA-B | HOXA9 |  |  |  |  |
| TBC1D1 | FOXD3 | PKD2 | FASTKD2 |  |  |  |  |
| DNAJA3 | PRG2 | ENG | FGF21 |  |  |  |  |
| NME6 | IL36RN | NOD2 | CSMD1 |  |  |  |  |
| NIF3L1 | IL18BP | NCF2 | OXA1L |  |  |  |  |
| LIN7A | PEX26 | CYBA | IQSEC2 |  |  |  |  |
| DYNC1I1 | TNIP1 | HLA-DRB1 | IL18RAP |  |  |  |  |
| TNNI1 | CXCL13 | IL10 | IFI27 |  |  |  |  |
| PCDH10 | BRF1 | CYBB | LYST |  |  |  |  |
| HIF3A | DMBT1 | YES1 | GPBAR1 |  |  |  |  |
| SLC15A2 | COQ2 | MAPKAPK2 | ITLN1 |  |  |  |  |
| RPL23 | CLEC1A | MAPK7 | DDX3Y |  |  |  |  |
| TRIP10 | S100A1 | RARG | RIN3 |  |  |  |  |
| COL8A1 | FREM1 | PRLR | ESCO2 |  |  |  |  |
| PDIA6 | KDM4C | FAAH | LMOD3 |  |  |  |  |
| ABCA6 | MRE11 | KLKB1 | RAB8A |  |  |  |  |
| DNER | TBL2 | TRPC3 | TCF20 |  |  |  |  |
| CA10 | MYPN | CDC25C | CEP41 |  |  |  |  |
| MSI1 | MICB | CASP2 | IL32 |  |  |  |  |
| SLC16A4 | TNFSF12 | SYT1 | DISP1 |  |  |  |  |
| CPEB1 | BAZ1B | CCNE1 | GLIS3 |  |  |  |  |
| CREB5 | SON | FPR1 | NOS1AP |  |  |  |  |
| CARD10 | MUC4 | SLC6A1 | FBXO38 |  |  |  |  |
| ARSF | IVNS1ABP | SLC29A1 | KMT2B |  |  |  |  |
| MMP25 | TCTN3 | ITGB6 | SETD5 |  |  |  |  |
| CER1 | RAI1 | DUSP6 | TMEM70 |  |  |  |  |
| USF2 | JMJD1C | CTBP1 | MYO9A |  |  |  |  |
| CATSPER1 | MRC1 | GRIK2 | PIP |  |  |  |  |
| RPL6 | IFT88 | USP7 | DYM |  |  |  |  |
| OLFM4 | ARHGAP31 | FZD2 | AIF1 |  |  |  |  |
| SRSF9 | NTS | SSTR2 | HYLS1 |  |  |  |  |
| ALDH1L1 | CD5 | KIF11 | PVALB |  |  |  |  |
| MGAT5 | SGCB | CSNK2B | LZTFL1 |  |  |  |  |
| C1GALT1 | PDPN | MAPK9 | PEX16 |  |  |  |  |
| RFWD3 | ATXN7 | TLR8 | PGAP3 |  |  |  |  |
| AGR2 | KRT20 | STIM1 | KANSL1 |  |  |  |  |
| FSTL3 | SYNE1 | SGK1 | MYOM2 |  |  |  |  |
| SEC14L2 | UTS2 | ATP1A2 | BBS5 |  |  |  |  |
| TLX1 | KLRK1 | CASK | BBS9 |  |  |  |  |
| OASL | SEMA3D | KCNJ2 | ANKRD11 |  |  |  |  |
| SLC24A3 | NPPC | ADI1 | UBAC2 |  |  |  |  |
| CDC5L | CEP164 | HSD17B12 | FRAS1 |  |  |  |  |
| AK7 | POGLUT1 | DIS3 | B9D2 |  |  |  |  |
| AMMECR1 | CAMP | LMO7 | OXT |  |  |  |  |
| ID4 | TK2 | KIF4A | TTC37 |  |  |  |  |
| SNRPD2 | BBS10 | UBR2 | BBS7 |  |  |  |  |
| G3BP1 | CALB2 | NR2C1 | FUZ |  |  |  |  |
| EHF | EBF3 | CEPT1 | DYNC2LI1 |  |  |  |  |
| SLC30A4 | GAST | EMB | SLC52A3 |  |  |  |  |
| REG4 | IL11 | MAST2 | TBCE |  |  |  |  |
| RSRC1 | DOCK6 | RAB5C | OTULIN |  |  |  |  |
| DNAL4 | IL22 | LIMS1 | PEX12 |  |  |  |  |
| RAB18 | PPIG | SETMAR | KLRC4 |  |  |  |  |
| SCG2 | IL16 | CERS2 | S100A12 |  |  |  |  |
| INSIG2 | CD69 | DOCK4 | CLIP2 |  |  |  |  |
| CTRL | STX11 | FUT9 | TCTN2 |  |  |  |  |
| SERPINA12 | SHOC2 | PCDH8 | ARVCF |  |  |  |  |
| PSMD8 | NALCN | INSIG1 | MUC16 |  |  |  |  |
| SLAMF6 | BLZF1 | RGS20 | MADCAM1 |  |  |  |  |
| ATOX1 | HEYL | CKS1B | HBD |  |  |  |  |
| FKBP1B | SRP72 | BTN1A1 | MLANA |  |  |  |  |
| PPY | FRMD7 | BHMT2 | AOC1 |  |  |  |  |
| CD160 | PRDM5 | RAP2A | MYOG |  |  |  |  |
| ABCF1 | CXCL16 | WASF3 | TTF2 |  |  |  |  |
| AOAH | CLC | TSN | C2CD3 |  |  |  |  |
| ALG11 | MARCKS | CDC27 | TNS1 |  |  |  |  |
| DACT1 | OSTM1 | ATAD3A | TRMU |  |  |  |  |
| RARRES2 | OTUD6B | BCL2A1 | PIGG |  |  |  |  |
| DAAM2 | CD207 | EMG1 | PLA2R1 |  |  |  |  |
| IL19 | CD83 | UTRN | CD99 |  |  |  |  |
| IL13RA2 | HEY2 | WAC | PQBP1 |  |  |  |  |
| SHANK3 | IFT122 | LSM2 | ADAMTSL4 |  |  |  |  |
| PRSS23 | MUC2 | SLC45A2 | MKRN3 |  |  |  |  |
| CD68 | FREM2 | KIF3B | TBX22 |  |  |  |  |
| CD58 | CEP120 | IFT57 | LPO |  |  |  |  |
| P3H1 | PRSS2 | CD7 | SPN |  |  |  |  |
| NES | PIGL | LAT2 | CD200 |  |  |  |  |
| BLVRB | SCGB3A2 | MYF5 | KLF11 |  |  |  |  |
| SIX5 | EVC2 | DEFB1 | RNASE2 |  |  |  |  |
| APC2 | RASA2 | RNF213 | CD1C |  |  |  |  |
| FAT4 | TTC12 | BTNL2 | AP3D1 |  |  |  |  |
| NDUFAF2 | RSPH1 | MED13L | TRIM71 |  |  |  |  |
| HSPA1B | DOK7 | BGLAP | FANCB |  |  |  |  |
| MAPKBP1 | NME8 | IFT172 | ARHGAP24 |  |  |  |  |
| MUC6 | TMCO1 | USH2A | YY1AP1 |  |  |  |  |
| KIF7 | GATA5 | CSF3 | RND3 |  |  |  |  |
| MAGT1 | DNAI2 | IFNA1 | PDE8A |  |  |  |  |
| GBX2 | SPG11 | CXCL8 | ATP6V1D |  |  |  |  |
| CCT6B | GIGYF2 | ANGPTL1 | GSTK1 |  |  |  |  |
| CDYL | MKKS | CHST5 | ZBTB17 |  |  |  |  |
| KDM4A | DCDC2 | SEC24B | LSR |  |  |  |  |
| VAMP3 | ICOSLG | RAB1A | CBLN1 |  |  |  |  |
