# Supplementary material for: Network Pharmacology and Experimental Verification Revealed the Mechanism of Yiqi Jianpi Recipe on Chronic Obstructive Pulmonary Disease
Source: Evid Based Complement Alternat Med. 2022 Sep 7;2022:8823231. doi: 10.1155/2022/8823231 (PMC9473879; doi:10.1155/2022/8823231)
Supplement: Supplementary Materials — Supplementary Table 1: information of collected YQJPR active ingredients. Supplementary Table 2: results of Venn diagram. Supplementary Table 3: data of KEGG enrichment analysis. [file 8823231.f1.zip › Supplementary Table 3.docx]

**Supplementary Table 3: data of KEGG enrichment analysis.**

| **ID** | **Description** | **geneID** |
| --- | --- | --- |
| hsa05167 | Kaposi sarcoma-associated herpesvirus infection | AKT1/IL6/JUN/VEGFA/CASP3/PTGS2/MYC/HIF1A/MAPK3/STAT3/FOS/CCND1/CREB1 |
| hsa05207 | Chemical carcinogenesis - receptor activation | AKT1/JUN/VEGFA/ESR1/EGFR/MYC/MAPK3/STAT3/EGF/HSP90AA1/FOS/CCND1/CREB1 |
| hsa04668 | TNF signaling pathway | AKT1/IL6/JUN/CASP3/IL1B/PTGS2/MAPK3/MMP9/FOS/CREB1/CCL2 |
| hsa05163 | Human cytomegalovirus infection | AKT1/IL6/VEGFA/CASP3/IL1B/PTGS2/EGFR/MYC/MAPK3/STAT3/CCND1/CREB1/CCL2 |
| hsa04657 | IL-17 signaling pathway | IL6/JUN/CASP3/IL1B/PTGS2/MAPK3/MMP9/HSP90AA1/FOS/CCL2 |
| hsa05205 | Proteoglycans in cancer | AKT1/VEGFA/CASP3/ESR1/EGFR/MYC/HIF1A/MAPK3/STAT3/MMP9/CCND1/ERBB2 |
| hsa04933 | AGE-RAGE signaling pathway in diabetic complications | AKT1/IL6/JUN/VEGFA/CASP3/IL1B/MAPK3/STAT3/CCND1/CCL2 |
| hsa05417 | Lipid and atherosclerosis | AKT1/IL6/JUN/CASP3/IL1B/MAPK3/STAT3/MMP9/PPARG/HSP90AA1/FOS/CCL2 |
| hsa05219 | Bladder cancer | VEGFA/EGFR/MYC/MAPK3/MMP9/EGF/CCND1/ERBB2 |
| hsa05210 | Colorectal cancer | AKT1/JUN/CASP3/EGFR/MYC/MAPK3/EGF/FOS/CCND1 |
| hsa05215 | Prostate cancer | AKT1/EGFR/MAPK3/MMP9/EGF/HSP90AA1/CCND1/ERBB2/CREB1 |
| hsa05224 | Breast cancer | AKT1/JUN/ESR1/EGFR/MYC/MAPK3/EGF/FOS/CCND1/ERBB2 |
| hsa01522 | Endocrine resistance | AKT1/JUN/ESR1/EGFR/MAPK3/MMP9/FOS/CCND1/ERBB2 |
| hsa05161 | Hepatitis B | AKT1/IL6/JUN/CASP3/MYC/MAPK3/STAT3/MMP9/FOS/CREB1 |
| hsa04066 | HIF-1 signaling pathway | AKT1/IL6/VEGFA/EGFR/HIF1A/MAPK3/STAT3/EGF/ERBB2 |
| hsa05212 | Pancreatic cancer | AKT1/VEGFA/EGFR/MAPK3/STAT3/EGF/CCND1/ERBB2 |
| hsa01521 | EGFR tyrosine kinase inhibitor resistance | AKT1/IL6/VEGFA/EGFR/MAPK3/STAT3/EGF/ERBB2 |
| hsa04915 | Estrogen signaling pathway | AKT1/JUN/ESR1/EGFR/MAPK3/MMP9/HSP90AA1/FOS/CREB1 |
| hsa05235 | PD-L1 expression and PD-1 checkpoint pathway in cancer | AKT1/JUN/EGFR/HIF1A/MAPK3/STAT3/EGF/FOS |
| hsa04010 | MAPK signaling pathway | AKT1/JUN/VEGFA/CASP3/IL1B/EGFR/MYC/MAPK3/EGF/FOS/ERBB2 |
| hsa05213 | Endometrial cancer | AKT1/EGFR/MYC/MAPK3/EGF/CCND1/ERBB2 |
| hsa04659 | Th17 cell differentiation | IL6/JUN/IL1B/HIF1A/MAPK3/STAT3/HSP90AA1/FOS |
| hsa04151 | PI3K-Akt signaling pathway | AKT1/IL6/VEGFA/EGFR/MYC/MAPK3/EGF/HSP90AA1/CCND1/ERBB2/CREB1 |
| hsa05223 | Non-small cell lung cancer | AKT1/EGFR/MAPK3/STAT3/EGF/CCND1/ERBB2 |
| hsa04926 | Relaxin signaling pathway | AKT1/JUN/VEGFA/EGFR/MAPK3/MMP9/FOS/CREB1 |
| hsa05162 | Measles | AKT1/IL6/JUN/CASP3/IL1B/STAT3/FOS/CCND1 |
| hsa05418 | Fluid shear stress and atherosclerosis | AKT1/JUN/VEGFA/IL1B/MMP9/HSP90AA1/FOS/CCL2 |
| hsa04012 | ErbB signaling pathway | AKT1/JUN/EGFR/MYC/MAPK3/EGF/ERBB2 |
| hsa05206 | MicroRNAs in cancer | VEGFA/CASP3/PTGS2/EGFR/MYC/MAPK3/STAT3/MMP9/CCND1/ERBB2 |
| hsa05160 | Hepatitis C | AKT1/CASP3/EGFR/MYC/MAPK3/STAT3/EGF/CCND1 |
| hsa05231 | Choline metabolism in cancer | AKT1/JUN/EGFR/HIF1A/MAPK3/EGF/FOS |
| hsa05142 | Chagas disease | AKT1/IL6/JUN/IL1B/MAPK3/FOS/CCL2 |
| hsa05132 | Salmonella infection | AKT1/IL6/JUN/CASP3/IL1B/MYC/MAPK3/HSP90AA1/FOS |
| hsa04917 | Prolactin signaling pathway | AKT1/ESR1/MAPK3/STAT3/FOS/CCND1 |
| hsa05230 | Central carbon metabolism in cancer | AKT1/EGFR/MYC/HIF1A/MAPK3/ERBB2 |
| hsa04380 | Osteoclast differentiation | AKT1/JUN/IL1B/MAPK3/PPARG/FOS/CREB1 |
| hsa04510 | Focal adhesion | AKT1/JUN/VEGFA/EGFR/MAPK3/EGF/CCND1/ERBB2 |
| hsa04068 | FoxO signaling pathway | AKT1/IL6/EGFR/MAPK3/STAT3/EGF/CCND1 |
| hsa05133 | Pertussis | IL6/JUN/CASP3/IL1B/MAPK3/FOS |
| hsa05135 | Yersinia infection | AKT1/IL6/JUN/IL1B/MAPK3/FOS/CCL2 |
| hsa05166 | Human T-cell leukemia virus 1 infection | AKT1/IL6/JUN/MYC/MAPK3/FOS/CCND1/CREB1 |
| hsa05208 | Chemical carcinogenesis - reactive oxygen species | AKT1/JUN/VEGFA/EGFR/HIF1A/MAPK3/EGF/FOS |
| hsa05226 | Gastric cancer | AKT1/EGFR/MYC/MAPK3/EGF/CCND1/ERBB2 |
| hsa05165 | Human papillomavirus infection | AKT1/VEGFA/CASP3/PTGS2/EGFR/MAPK3/EGF/CCND1/CREB1 |
| hsa05171 | Coronavirus disease - COVID-19 | IL6/JUN/IL1B/EGFR/MAPK3/STAT3/FOS/CCL2 |
| hsa04932 | Non-alcoholic fatty liver disease | AKT1/IL6/JUN/CASP3/IL1B/PPARG/FOS |
| hsa05323 | Rheumatoid arthritis | IL6/JUN/VEGFA/IL1B/FOS/CCL2 |
| hsa04630 | JAK-STAT signaling pathway | AKT1/IL6/EGFR/MYC/STAT3/EGF/CCND1 |
| hsa04620 | Toll-like receptor signaling pathway | AKT1/IL6/JUN/IL1B/MAPK3/FOS |
| hsa04625 | C-type lectin receptor signaling pathway | AKT1/IL6/JUN/IL1B/PTGS2/MAPK3 |
| hsa04919 | Thyroid hormone signaling pathway | AKT1/ESR1/MYC/HIF1A/MAPK3/CCND1 |
| hsa05169 | Epstein-Barr virus infection | AKT1/IL6/JUN/CASP3/MYC/STAT3/CCND1 |
| hsa05221 | Acute myeloid leukemia | AKT1/MYC/MAPK3/STAT3/CCND1 |
| hsa05211 | Renal cell carcinoma | AKT1/JUN/VEGFA/HIF1A/MAPK3 |
| hsa05218 | Melanoma | AKT1/EGFR/MAPK3/EGF/CCND1 |
| hsa05214 | Glioma | AKT1/EGFR/MAPK3/EGF/CCND1 |
| hsa05140 | Leishmaniasis | JUN/IL1B/PTGS2/MAPK3/FOS |
| hsa04921 | Oxytocin signaling pathway | JUN/PTGS2/EGFR/MAPK3/FOS/CCND1 |
| hsa05216 | Thyroid cancer | MYC/MAPK3/PPARG/CCND1 |
| hsa05222 | Small cell lung cancer | AKT1/CASP3/PTGS2/MYC/CCND1 |
| hsa05164 | Influenza A | AKT1/IL6/CASP3/IL1B/MAPK3/CCL2 |
| hsa05152 | Tuberculosis | AKT1/IL6/CASP3/IL1B/MAPK3/CREB1 |
| hsa04621 | NOD-like receptor signaling pathway | IL6/JUN/IL1B/MAPK3/HSP90AA1/CCL2 |
| hsa05130 | Pathogenic Escherichia coli infection | IL6/JUN/CASP3/IL1B/MAPK3/FOS |
| hsa05203 | Viral carcinogenesis | JUN/CASP3/MAPK3/STAT3/CCND1/CREB1 |
| hsa04935 | Growth hormone synthesis, secretion and action | AKT1/MAPK3/STAT3/FOS/CREB1 |
| hsa04370 | VEGF signaling pathway | AKT1/VEGFA/PTGS2/MAPK3 |
| hsa05321 | Inflammatory bowel disease | IL6/JUN/IL1B/STAT3 |
| hsa04210 | Apoptosis | AKT1/JUN/CASP3/MAPK3/FOS |
| hsa04936 | Alcoholic liver disease | AKT1/IL6/CASP3/IL1B/CCND1 |
| hsa01524 | Platinum drug resistance | AKT1/CASP3/MAPK3/ERBB2 |
| hsa05220 | Chronic myeloid leukemia | AKT1/MYC/MAPK3/CCND1 |
| hsa04218 | Cellular senescence | AKT1/IL6/MYC/MAPK3/CCND1 |
| hsa04662 | B cell receptor signaling pathway | AKT1/JUN/MAPK3/FOS |
| hsa05225 | Hepatocellular carcinoma | AKT1/EGFR/MYC/MAPK3/CCND1 |
| hsa04660 | T cell receptor signaling pathway | AKT1/JUN/MAPK3/FOS |
| hsa04928 | Parathyroid hormone synthesis, secretion and action | EGFR/MAPK3/FOS/CREB1 |
| hsa04931 | Insulin resistance | AKT1/IL6/STAT3/CREB1 |
| hsa04015 | Rap1 signaling pathway | AKT1/VEGFA/EGFR/MAPK3/EGF |
| hsa05145 | Toxoplasmosis | AKT1/CASP3/MAPK3/STAT3 |
| hsa05170 | Human immunodeficiency virus 1 infection | AKT1/JUN/CASP3/MAPK3/FOS |
| hsa04725 | Cholinergic synapse | AKT1/MAPK3/FOS/CREB1 |
| hsa04024 | cAMP signaling pathway | AKT1/JUN/MAPK3/FOS/CREB1 |
| hsa04152 | AMPK signaling pathway | AKT1/PPARG/CCND1/CREB1 |
| hsa05144 | Malaria | IL6/IL1B/CCL2 |
| hsa04014 | Ras signaling pathway | AKT1/VEGFA/EGFR/MAPK3/EGF |
| hsa05010 | Alzheimer disease | AKT1/IL6/CASP3/IL1B/PTGS2/MAPK3 |
| hsa05131 | Shigellosis | AKT1/JUN/IL1B/EGFR/MAPK3 |
| hsa05134 | Legionellosis | IL6/CASP3/IL1B |
| hsa04550 | Signaling pathways regulating pluripotency of stem cells | AKT1/MYC/MAPK3/STAT3 |
| hsa04072 | Phospholipase D signaling pathway | AKT1/EGFR/MAPK3/EGF |
| hsa05020 | Prion disease | IL6/CASP3/IL1B/MAPK3/CREB1 |
| hsa04934 | Cushing syndrome | EGFR/MAPK3/CCND1/CREB1 |
| hsa05031 | Amphetamine addiction | JUN/FOS/CREB1 |
| hsa05120 | Epithelial cell signaling in Helicobacter pylori infection | JUN/CASP3/EGFR |
| hsa04520 | Adherens junction | EGFR/MAPK3/ERBB2 |
| hsa04062 | Chemokine signaling pathway | AKT1/MAPK3/STAT3/CCL2 |
| hsa05202 | Transcriptional misregulation in cancer | IL6/MYC/MMP9/PPARG |
| hsa04540 | Gap junction | EGFR/MAPK3/EGF |
| hsa04211 | Longevity regulating pathway | AKT1/PPARG/CREB1 |
| hsa04658 | Th1 and Th2 cell differentiation | JUN/MAPK3/FOS |
| hsa04912 | GnRH signaling pathway | JUN/EGFR/MAPK3 |
| hsa04713 | Circadian entrainment | MAPK3/FOS/CREB1 |
| hsa04914 | Progesterone-mediated oocyte maturation | AKT1/MAPK3/HSP90AA1 |
| hsa05146 | Amoebiasis | IL6/CASP3/IL1B |
| hsa01523 | Antifolate resistance | IL6/IL1B |
| hsa04726 | Serotonergic synapse | CASP3/PTGS2/MAPK3 |
| hsa04020 | Calcium signaling pathway | VEGFA/EGFR/EGF/ERBB2 |
| hsa04722 | Neurotrophin signaling pathway | AKT1/JUN/MAPK3 |
| hsa05143 | African trypanosomiasis | IL6/IL1B |
| hsa04728 | Dopaminergic synapse | AKT1/FOS/CREB1 |
| hsa05332 | Graft-versus-host disease | IL6/IL1B |
| hsa04371 | Apelin signaling pathway | AKT1/MAPK3/CCND1 |
| hsa04140 | Autophagy - animal | AKT1/HIF1A/MAPK3 |
| hsa04261 | Adrenergic signaling in cardiomyocytes | AKT1/MAPK3/CREB1 |
| hsa05030 | Cocaine addiction | JUN/CREB1 |
| hsa05022 | Pathways of neurodegeneration - multiple diseases | IL6/CASP3/IL1B/PTGS2/MAPK3 |
| hsa04217 | Necroptosis | IL1B/STAT3/HSP90AA1 |
| hsa05168 | Herpes simplex virus 1 infection | AKT1/IL6/CASP3/IL1B/CCL2 |
| hsa04022 | cGMP-PKG signaling pathway | AKT1/MAPK3/CREB1 |
| hsa04310 | Wnt signaling pathway | JUN/MYC/CCND1 |
| hsa04923 | Regulation of lipolysis in adipocytes | AKT1/PTGS2 |
| hsa04530 | Tight junction | JUN/CCND1/ERBB2 |
| hsa05416 | Viral myocarditis | CASP3/CCND1 |
| hsa04623 | Cytosolic DNA-sensing pathway | IL6/IL1B |
| hsa04929 | GnRH secretion | AKT1/MAPK3 |
| hsa04664 | Fc epsilon RI signaling pathway | AKT1/MAPK3 |
| hsa04920 | Adipocytokine signaling pathway | AKT1/STAT3 |
| hsa04137 | Mitophagy - animal | JUN/HIF1A |
| hsa04115 | p53 signaling pathway | CASP3/CCND1 |
| hsa04612 | Antigen processing and presentation | HSP90AA1/CREB1 |
| hsa04810 | Regulation of actin cytoskeleton | EGFR/MAPK3/EGF |
| hsa04350 | TGF-beta signaling pathway | MYC/MAPK3 |
| hsa04666 | Fc gamma R-mediated phagocytosis | AKT1/MAPK3 |
| hsa04640 | Hematopoietic cell lineage | IL6/IL1B |
| hsa04061 | Viral protein interaction with cytokine and cytokine receptor | IL6/CCL2 |
| hsa04916 | Melanogenesis | MAPK3/CREB1 |
| hsa04064 | NF-kappa B signaling pathway | IL1B/PTGS2 |
| hsa04922 | Glucagon signaling pathway | AKT1/CREB1 |
